# Targeting *Peptostreptococcus anaerobius* with an Iron‐Based Nanozyme Reverses Ferroptosis Resistance and Enhances Antitumor Immunity in Colorectal Cancer

**DOI:** 10.1002/advs.202516272

**Published:** 2026-01-12

**Authors:** Yinghao Cao, Jun Wang, Hanwenchen Wang, Xiang Sun, Fang Fang, Shiying Li, Jianping Liu, Changrong Shi, Pengyuan Qi, Jianhua Zou, Xiaoyuan Chen, Kailin Cai

**Affiliations:** ^1^ Department of Digestive Surgical Oncology Union Hospital Tongji Medical College Huazhong University of Science and Technology Wuhan China; ^2^ Cancer Center Union Hospital Tongji Medical College Huazhong University of Science and Technology Wuhan Hubei China; ^3^ Departments of Diagnostic Radiology Yong Loo Lin School of Medicine and College of Design and Engineering National University of Singapore Singapore Singapore; ^4^ Nanomedicine Translational Research Program Yong Loo Lin School of Medicine National University of Singapore Singapore Singapore; ^5^ Department of Thoracic Surgery Union Hospital Tongji Medical College Huazhong University of Science and Technology Wuhan Hubei China; ^6^ Hubei Key Laboratory of Biological Targeted Therapy Union Hospital Tongji Medical College Huazhong University of Science and Technology Wuhan Hubei China; ^7^ Department of Gastrointestinal Surgery Union Hospital Tongji Medical College Huazhong University of Science and Technology Wuhan Hubei China; ^8^ Department of Chemical and Biomolecular Engineering College of Design and Engineering National University of Singapore Singapore Singapore; ^9^ Department of Biomedical Engineering College of Design and Engineering National University of Singapore Singapore Singapore; ^10^ Department of Pharmacy and Pharmaceutical Sciences Faculty of Science National University of Singapore Singapore Singapore; ^11^ Clinical Imaging Research Centre Centre for Translational Medicine Yong Loo Lin School of Medicine National University of Singapore Singapore Singapore; ^12^ Theranostics Center of Excellence (TCE) Yong Loo Lin School of Medicine National University of Singapore Singapore Singapore

**Keywords:** colorectal cancer, ferroptosis, immunotherapy, nanozyme, *P. anaerobius*

## Abstract

The intratumoral microbiota is a critical determinant of therapeutic outcomes in colorectal cancer (CRC). Although several microbial species have been identified that influence CRC development and treatment resistance, effective strategies for precisely modulating these bacteria remain limited. In this study, we identify *Peptostreptococcus anaerobius* as a tumor‐enriched anaerobe that promotes CRC progression by inhibiting ferroptosis. To counteract this, we engineered a composite iron‐based nanozyme encapsulating lincomycin, which selectively targets and eradicates intratumoral *P. anaerobius*, thereby reversing ferroptosis resistance in CRC. Simultaneously, the nanozyme's inherent peroxidase (POD) like activity catalyzes hydroxyl radical generation, enhancing intracellular oxidative stress and promoting ferroptosis. This dual mechanism‐microbial clearance and ROS‐mediated ferroptosis induction‐synergistically suppresses tumor growth. Moreover, ferroptotic cancer cells release large amounts of damage‐associated molecular patterns (DAMPs), which trigger immunogenic cell death (ICD), promoting dendritic cell (DC) maturation and T cell activation, thereby enhancing anti‐tumor immunity. Our findings highlight a novel ferroptosis‐centered therapeutic strategy integrating microbiota modulation and catalytic nanomedicine for precise CRC treatment.

## Graphical abstract

An iron‐based nanozyme selectively eliminates intratumoral *P. anaerobius* while catalytically generating ROS to induce ferroptosis, synergistically suppressing colorectal cancer growth and activating anti‐tumor immunity through immunogenic cell death.

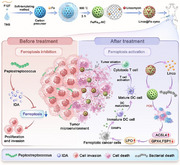

## 1 Introduction

CRC is a highly prevalent malignancy worldwide, ranking among the leading causes of cancer‐related incidence and mortality [1, 2]. Despite considerable progress in early detection, surgical techniques, and systemic therapies, overall prognosis, particularly for advanced‐stage CRC remains unsatisfactory [3, 4]. CRC therapy encounters multiple obstacles, such as increasing resistance to standard chemotherapy and radiotherapy, limited effectiveness of targeted therapies, and inadequate responses to immunotherapy [5]. Ferroptosis, an iron‐dependent form of regulated cell death characterized by lipid peroxidation (LPO), has recently gained attention as a potential avenue for cancer treatment [6, 7]. However, tumor cells can evade ferroptotic death by reprogramming metabolic and redox pathways, thereby enhancing their survival [8, 9]. Notably, certain anaerobic bacteria modulate iron metabolism and oxidative stress responses, thereby interfering with ferroptotic signaling and undermining therapeutic efficacy [10, 11, 12]. Intratumoral microbiota such as *P. anaerobius* have been implicated in ferroptosis suppression [13]. The tryptophan metabolite indole‐3‐acrylic acid (IDA), produced by *P. anaerobius*, has been shown to inhibit ferroptosis through FSP1‐mediated mechanisms [14, 15]. Thus, targeting the overabundance of *P. anaerobius* in CRC may represent a potential therapeutic strategy.

Growing evidence underscores the pivotal role of the tumor microenvironment (TME) in CRC progression, with the intratumoral microbiota emerging as a key component [16, 17]. Recent studies have revealed that specific microbial taxa are enriched in CRC tissues and actively contribute to tumorigenesis through diverse mechanisms, including the induction of chronic inflammation, production of genotoxic metabolite [18, 19], disruption of epithelial barrier integrity, and modulation of host immune responses [20, 21]. Furthermore, certain microbes have been shown to alter the tumor's metabolic landscape and influence the efficacy of chemotherapies, targeted therapies, and immunotherapies, thereby promoting therapeutic resistance [22, 23, 24]. Eliminating these pathogenic bacteria could help suppress tumor growth and progression [25]. Consequently, strategies aimed at precisely regulating the intratumoral microbiota have garnered increasing attention and represent a promising direction for cancer therapy.

Over the past few decades, targeted cancer therapies have seen unprecedented advances, and the development of nanomaterial‐based drug delivery platforms has made precision modulation of the intratumoral microbiota increasingly feasible [26]. Nevertheless, conventional intravenous antibiotic therapy is insufficient to effectively suppress intratumoral microbiota, leading to suboptimal therapeutic outcomes for tumor treatment [27]. Therefore, how to precisely deliver antibiotics to tumor sites remains a critical issue for inhibiting CRC progression. Single‐atom nanozymes (SAEs), characterized by atomically dispersed active metal sites, mimic the catalytic centers of natural enzymes and demonstrate superior enzymatic activity [28, 29, 30]. In addition to their inherent catalytic functions, SAEs offer controllable synthesis, high stability, and porous surfaces that make them suitable as drug delivery vehicles for various clinical applications [31, 32, 33]. Therefore, the combination of SAE‐based drug delivery and microbiome regulation offers an effective strategy for targeted tumor therapy [34, 35]. Fe‐SAE, with their excellent porosity, can serve as efficient drug delivery carriers for targeted therapy [36, 37]. Moreover, Fe‐SAE exhibit remarkable POD‐like activity, generating large amounts of ROS, such as hydroxyl radicals, within the TME [38]. This overwhelms the cellular antioxidant defense system and collapses redox homeostasis, ultimately facilitating ferroptosis.

**SCHEME 1:**
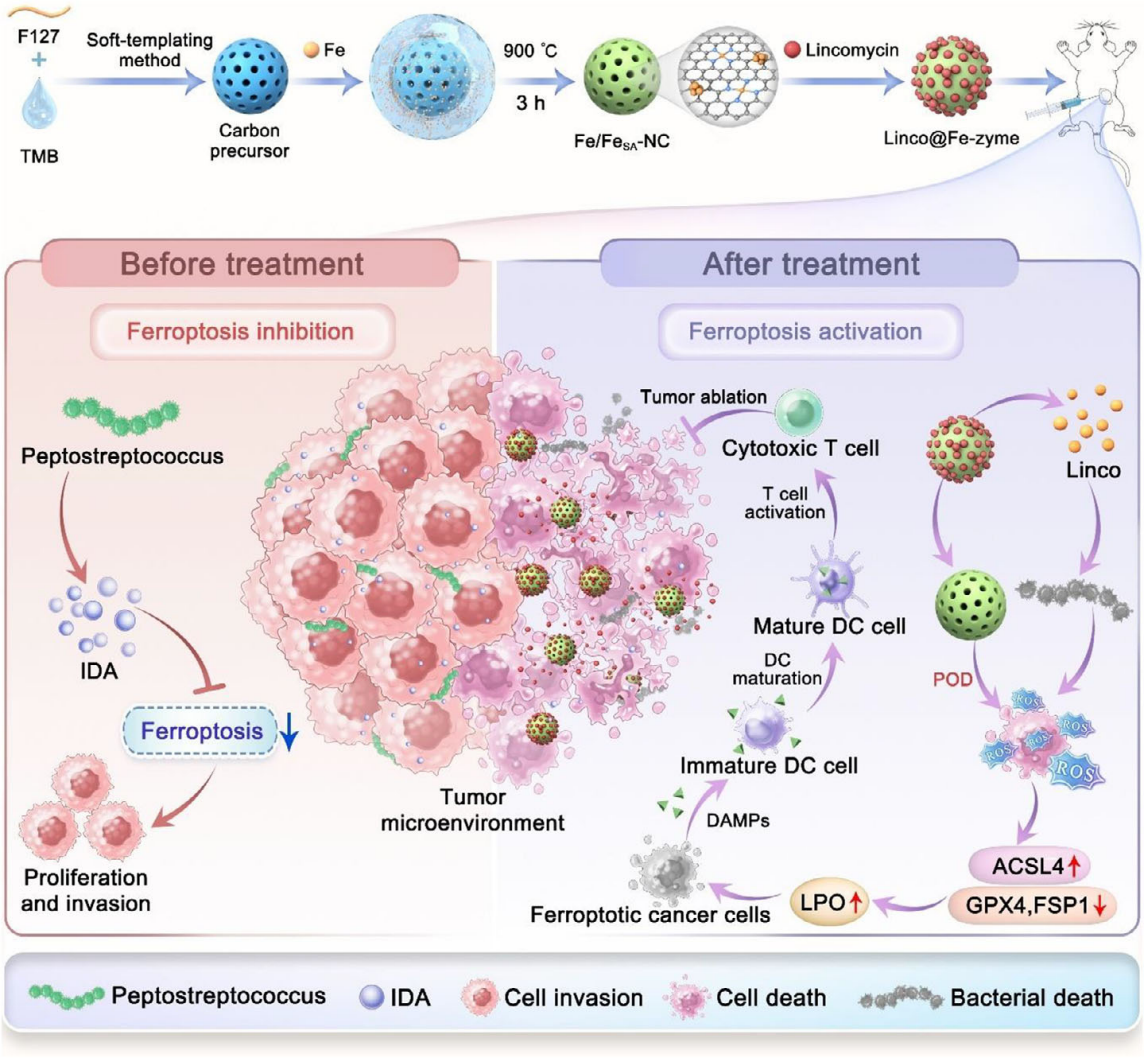
Schematic illustration of CRC therapy via Linco@Fe‐zyme targeting *P. anaerobius* to reverse ferroptosis resistance and trigger ICD.

In this study, we present a Fe‐SAE‐based delivery system that incorporates lincomycin, a selective antibiotic targeting anaerobic bacteria, into an iron‐based nanozyme platform. This system enables the targeted delivery of lincomycin to the TME, where it is released to eliminate *P. anaerobius*, an intratumoral bacterium known to suppress ferroptosis in CRC. Concurrently, the Fe‐SAE exhibits potent POD‐like catalytic activity, generating substantial levels of ROS. The combined antibacterial and catalytic effects synergistically induce ferroptosis in CRC cells by promoting lipid peroxidation (LPO), thereby effectively inhibiting tumor progression. Moreover, ferroptotic cancer cells release large amounts of DAMPs, which trigger ICD, promoting DC maturation and T cell activation, thereby enhancing anti‐tumor immunity (Scheme 1). Collectively, these mechanisms work in synergy to trigger ferroptosis in CRC cells and suppress tumor growth. This study not only presents a novel therapeutic approach for colorectal cancer but also provides a scientific basis for the future development of microbiota‐targeted anti‐cancer treatments.

This study reveals a critical role of intratumoral microbiota in regulating ferroptosis within the CRC microenvironment. Previous studies have linked gut microbiota to the malignant progression of CRC [39, 40]. Notably, the tryptophan metabolite IDA, derived from *P. anaerobius*, is closely associated with FSP1‐mediated ferroptosis inhibition [14, 15]. Therefore, targeting the overabundance of *P. anaerobius* in CRC may represent a novel and promising therapeutic approach.

## 2 Results

### 2.1 *P. anaerobius* is Abundant in CRC and Promotes Tumor Progression by Inhibiting Ferroptosis

**FIGURE 1:**
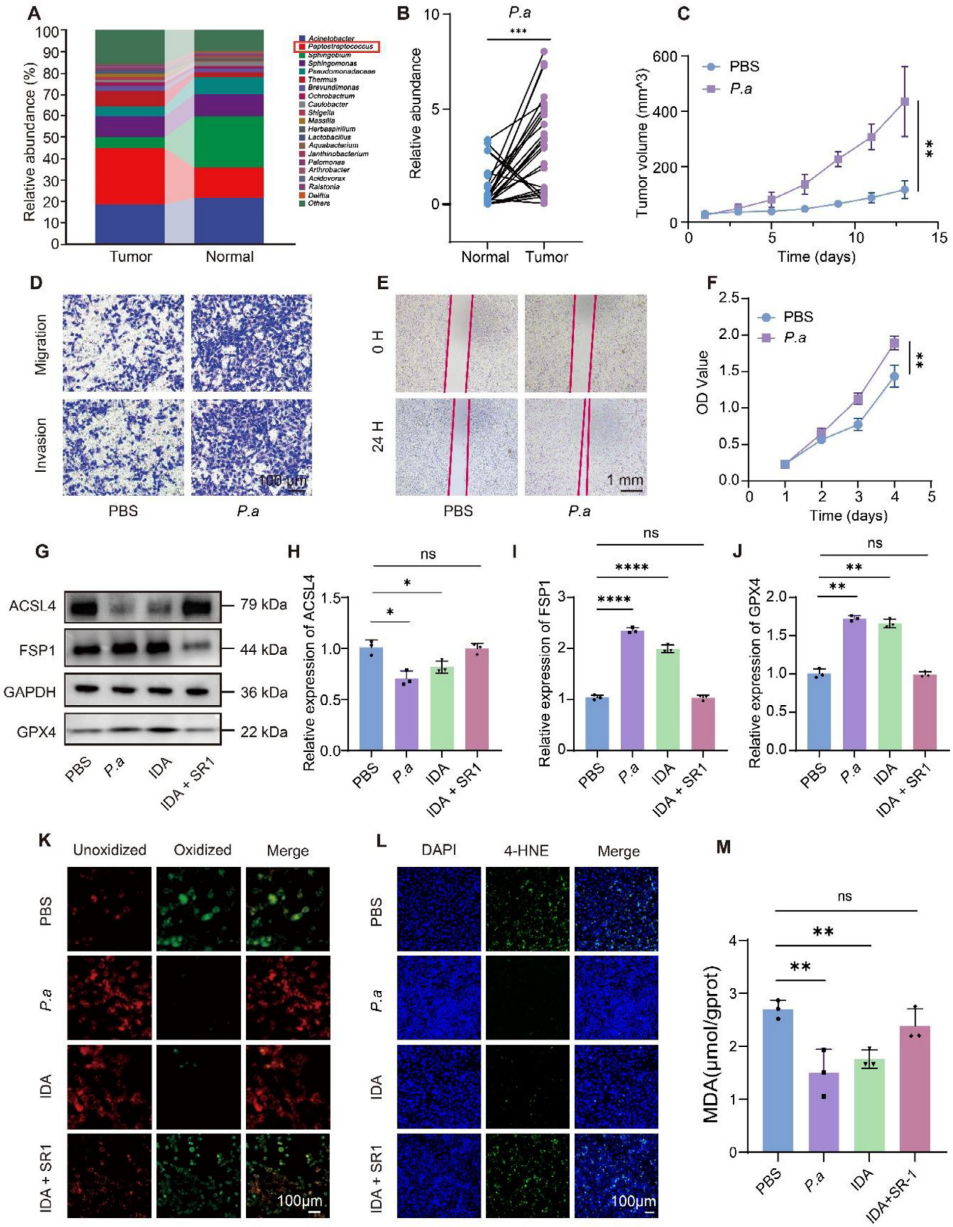
*P. anaerobius* is highly enriched in CRC and promotes tumor progression by inhibiting ferroptosis. (A) 16S rRNA sequencing showing increased abundance of *P. anaerobius* in CRC tumors compared to adjacent normal tissues. (B). Quantification of *P. anaerobius* abundance across groups (*n* = 25 paired tumor and adjacent normal tissues). Paired t‐test analysis indicated that the abundance of *P. anaerobius* was significantly higher in tumor tissues compared with adjacent normal tissues. (C). Tumor growth curves showing accelerated growth in the *P. anaerobius*‐treated group. (D). Transwell assays indicating enhanced CRC cell migration and invasion following *P. anaerobius* treatment, magnification = ×200. (E). Wound healing assays showing increased migration after *P. anaerobius* exposure, magnification = ×100. (F). CCK‐8 assay demonstrating increased proliferation of CRC cells upon *P. anaerobius* treatment. (G). Western blot analysis showing ferroptosis inhibition in CRC cells treated with PBS, *P. anaerobius*, IDA, or IDA + SR1. *P. anaerobius* and IDA suppressed ferroptosis markers, while SR1 restored ferroptotic signaling. (H–J). qPCR results further confirming that *P. anaerobius* and IDA inhibit ferroptosis, which can be reversed by AHR blockade with SR1. (K). C11‐BODIPY fluorescence analysis of lipid peroxidation levels in different treatment groups; red fluorescence indicates non‐oxidized lipids, while green fluorescence indicates elevated lipid peroxidation. (L). Levels of the lipid peroxidation product 4‐HNE in different treatment groups. (M). Levels of the lipid peroxidation product MDA in different treatment groups. *p < 0.05, **p < 0.01, ***p < 0.001, ****p < 0.0001, ns = no significant difference.

The gut microbiota is critically involved in the initiation and progression of CRC [41]. To investigate microbial composition, we collected tumor and adjacent normal tissues from CRC patients and performed 16S rRNA sequencing (*n* = 25). The results showed a significant enrichment of *P. anaerobius* within tumor tissues (Figure 1A,B). To evaluate whether *P. anaerobius* promotes tumor growth, LoVo cells were used to establish a subcutaneous xenograft model in nude mice. To ensure that the bacterial abundance in the animal models accurately reflected the levels observed in clinical tumor tissues, we systematically tested different bacterial dosing strategies and treatment durations in both the subcutaneous and AOM/DSS orthotopic tumor models. In the AOM/DSS model, oral gavage of 10^5 CFU of *P. anaerobius* suspension per dose twice per week for two consecutive weeks resulted in intratumoral bacterial loads that were comparable to those detected in human colorectal tumor samples, as confirmed by PCR analysis (Figure S1A). In the subcutaneous tumor model, administration of 10^5 CFU *P. anaerobius* twice per week achieved a similar clinically relevant intratumoral abundance within 1 week (Figure S1B).

Next, following the optimized dosing strategy described above, once the subcutaneous tumors reached approximately 50 mm^3^, *P. anaerobius* suspension (50 µL) was injected intratumorally and peritumorally every other day for two doses. Tumor volumes were recorded every 2 days for 2 weeks. Mice treated with *P. anaerobius* developed visibly larger tumors compared to the PBS control group (Figure S2A), The tumor weight of the *P. anaerobius*‐treated group was significantly higher than that of the PBS control group (Figure S2B), confirming accelerated progression in the *P. anaerobius*‐treated group (Figure 1C). Importantly, body weight monitoring over 15 days revealed no significant differences between groups (Figure S2C), indicating that *P. anaerobius* enhanced tumor growth without compromising general health. Additionally, in an AOM/DSS‐induced orthotopic CRC model, the H&E staining of the mouse intestinal tissue indicated the successful construction of the AOM/DSS tumor (Figure S2D,E), *P. anaerobius* exposed mice developed a greater number of tumors (Figure S2F,G). To complement the in vivo findings, in vitro transwell and wound healing assays were performed. The results demonstrated that *P. anaerobius* promoted the invasion and migration of LoVo cells (Figure 1D,E), The quantitative results of Transwell assay further confirmed that *P. anaerobius* could promote the migration and invasion of CRC cells (Figure S2H,I). While CCK‐8 assays confirmed that *P. anaerobius* enhanced loVo cell proliferation (Figure 1F). However, the underlying mechanism of *P. anaerobius*‐induced tumor promotion remained unclear.

It has been reported that *P. anaerobius* may inhibit tumor cell ferroptosis by producing indole‐3‐acrylic acid (IDA), a tryptophan‐derived microbial metabolite [15]. By analyzing the gut microbiota metabolites detected by 16S sequencing, IDA, a metabolite of *P. anaerobius*, was abnormally increased (Figure S3A,B). Moreover, metabolomic profiling revealed that *P. anaerobius*‐derived metabolites are closely associated with glutamine metabolism (Figure S3C), which is an important metabolic pathway during ferroptosis [42]. IDA has been reported to inhibit ferroptosis in CRC cells via activation of the aryl hydrocarbon receptor (AHR) [14]. To verify whether *P. anaerobius* inhibits ferroptosis, LoVo cells were treated with live *P. anaerobius* and purified IDA, respectively, and followed by assessment of ferroptosis signaling in the presence or absence of the AHR inhibitor SR1. Western blot analysis revealed that the expression levels of ferroptosis markers showed suppression of Acyl‐CoA Synthetase Long Chain Family Member 4 (ACSL4) and upregulation of Glutathione Peroxidase 4 (GPX4) and Ferroptosis Suppressor Protein 1 (FSP1) following treatment with *P. anaerobius* and IDA, while SR1 reversed this effect (Figure 1G). Quantitative protein analysis confirmed the result of the Western Blot (Figure S4A–C). In addition, real‐time quantitative PCR also showed that both *P. anaerobius* and IDA suppressed ferroptosis (Figure 1H–J).

Considering that lipid peroxidation is one of the most direct indicators of ferroptosis, we examined the levels of lipid peroxidation products in LoVo cells under different treatments. The C11‐BODIPY fluorescent probe was used to assess intracellular lipid peroxidation. In its reduced state, C11‐BODIPY emits red fluorescence, whereas upon oxidation during lipid peroxidation, it shifts to green fluorescence. As shown in Figure 1K and Figure S4D,E, *P. anaerobius* and its metabolite IDA reduced the production of lipid peroxidation products by inhibiting ferroptosis. Treatment with SR1 reversed this phenomenon and restored lipid peroxidation levels. Similarly, measurements of the lipid peroxidation products 4‐hydroxy‐2‐nonenal (4‐HNE) and malondialdehyde (MDA) yielded consistent results (Figure 1L,M and Figure S4F), indicating that *P. anaerobius* and IDA act as key inhibitors of ferroptosis in CRC. Following the establishment of the ferroptosis‐inhibitory effects of *P. anaerobius* and IDA on tumor cells, we subsequently investigated whether inhibition of IDA signaling could attenuate tumor cell proliferation and invasion. Transwell assays demonstrated that both *P. anaerobius* and IDA promoted tumor cell migration and invasion, whereas SR1 effectively suppressed these processes (Figure S4G–I). These findings suggest that eliminating *P. anaerobius* may help inhibit CRC cell migration and invasion.

### 2.2 Screening of Antibiotics for Antitumor Activity Against *P. anaerobius*

**FIGURE 2:**
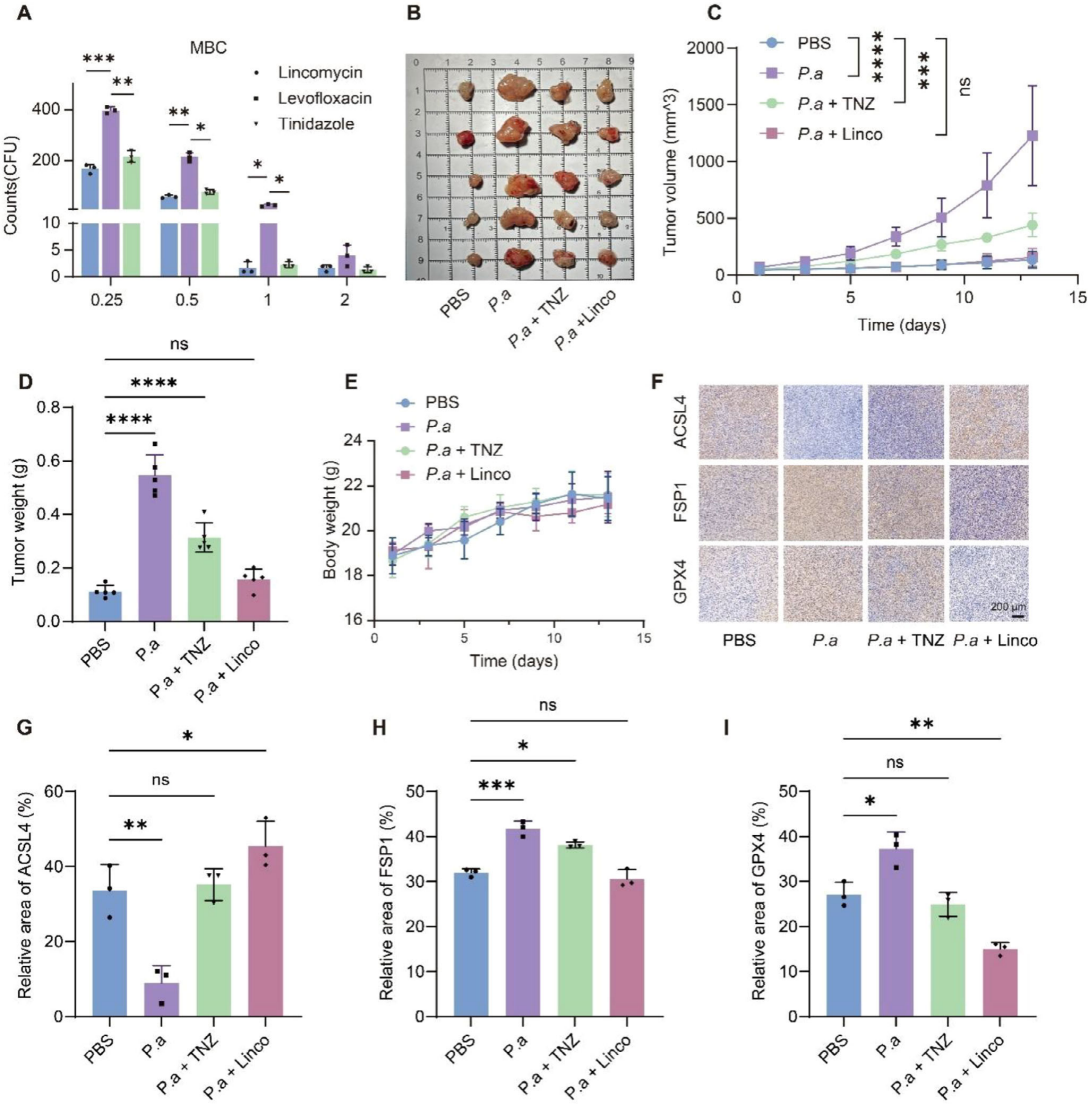
Linco promotes ferroptosis by clearing *P. anaerobius* from tumor tissues. (A). MBC assay of the three antibiotics with the lowest MIC values, indicating that lincomycin and Tinidazole exhibits the excellent MBC among the tested agents. (B). Gross images of subcutaneous tumors in mice treated with different antibiotics post *P. anaerobius* stimulation, showing optimal tumor suppression with Linco. (C). Tumor growth curves of mice across treatment groups, indicating robust inhibition by Linco. (D). Tumor mass comparison across groups. (E). Body weight changes over 2 weeks, confirming biosafety of the antibiotic treatments. (F). IHC analysis of tumor sections showing Linco‐enhanced ferroptosis. (G, H, I). Quantification of IHC staining for ACSL4, FSP1, and GPX4, supporting Linco‐mediated restoration of ferroptosis. *p < 0.05, **p < 0.01, ***p < 0.001, ****p < 0.0001, ns = no significant difference.

Modulating the composition of intratumoral gut microbiota has been recognized as a promising strategy for controlling tumor progression [43, 44]. Microbiota‐targeted therapies have been reported in multiple studies [45, 46]. Given the tumor‐promoting role of *P. anaerobius* in CRC, we hypothesized that selective elimination of intratumoral *P. anaerobius* might suppress CRC development. We evaluated the antimicrobial and bactericidal activities of several clinically relevant antibiotic classes, including macrolides (Azithromycin), lincosamides (Lincomycin), tetracyclines (Tetracycline), amphenicols (Chloramphenicol), and broad‐spectrum agents such as fluoroquinolones (Levofloxacin) and β‐lactams (Penicillin, cephalosporins). The Minimum Inhibitory Concentration (MIC) assay results showed that lincomycin (Linco), Penicillin, Tinidazole (TNZ), and Levofloxacin (LEV) exhibited lower than those of the other candidates the other candidates (Figure S5A). We then examined the Minimum Bactericidal Concentration (MBC) values of these four antibiotics. During this process, *P. anaerobius* acquired resistance; therefore, Penicillin was excluded as a viable option. Among the remaining three antibiotics, Linco and TNZ demonstrated superior bactericidal activity at the lowest concentration, producing significantly fewer surviving colonies than Levofloxacin (Figure 2A and Figure S5C). In addition, we collected *P. anaerobius* samples at different time points following lincomycin treatment and continued culturing them to assess residual growth capacity. The results showed that after treatment with 1 µg/mL lincomycin for 12 h, the bacteria had essentially lost their ability to grow (Figure S5B). Subsequently, we further evaluated the in vivo antibacterial and antitumor efficacy of these antibiotics.

To this end, we established subcutaneous tumor models in mice and administered either *P. anaerobius* or PBS via oral gavage. Mice with *P. anaerobius* induced tumors were then treated with Linco or TNZ. Compared to the group only treated with *P. anaerobius*, both antibiotic treatments significantly slowed tumor growth, with Linco demonstrating the most potent tumor‐suppressive effect (Figure 2B). Tumor volume assessments over a 2‐week period further validated that Linco treatment effectively abrogated the tumor‐promoting effect of *P. anaerobius* (Figure 2C). Consistently, tumor weights demonstrated the most pronounced reduction in the Linco‐treated group (Figure 2D). No significant difference in body weight was observed across groups, indicating minimal systemic toxicity from antibiotic administration (Figure 2E). To test whether the treatment of *P. anaerobius* with Linco or TNZ antibiotics improved ferroptosis of tumor cells, we collected different groups of tumor samples for immunohistochemical (IHC) study. Histological analysis of tumor tissues revealed that Linco‐treated tumors exhibited increased expression of the ferroptosis marker ACSL4, and decreased expression of ferroptosis inhibitors GPX4 and FSP1 (Figure 2F–I). Collectively, the results confirm that Linco effectively eradicated *P. anaerobius* from the TME, thereby relieving ferroptosis suppression, enhancing ferroptotic cell death in CRC cells, and ultimately inhibiting tumor progression.

### 2.3 Rationale for Targeted Lincomycin Delivery and Synthesis of Linco@Fe‐zyme

**FIGURE 3:**
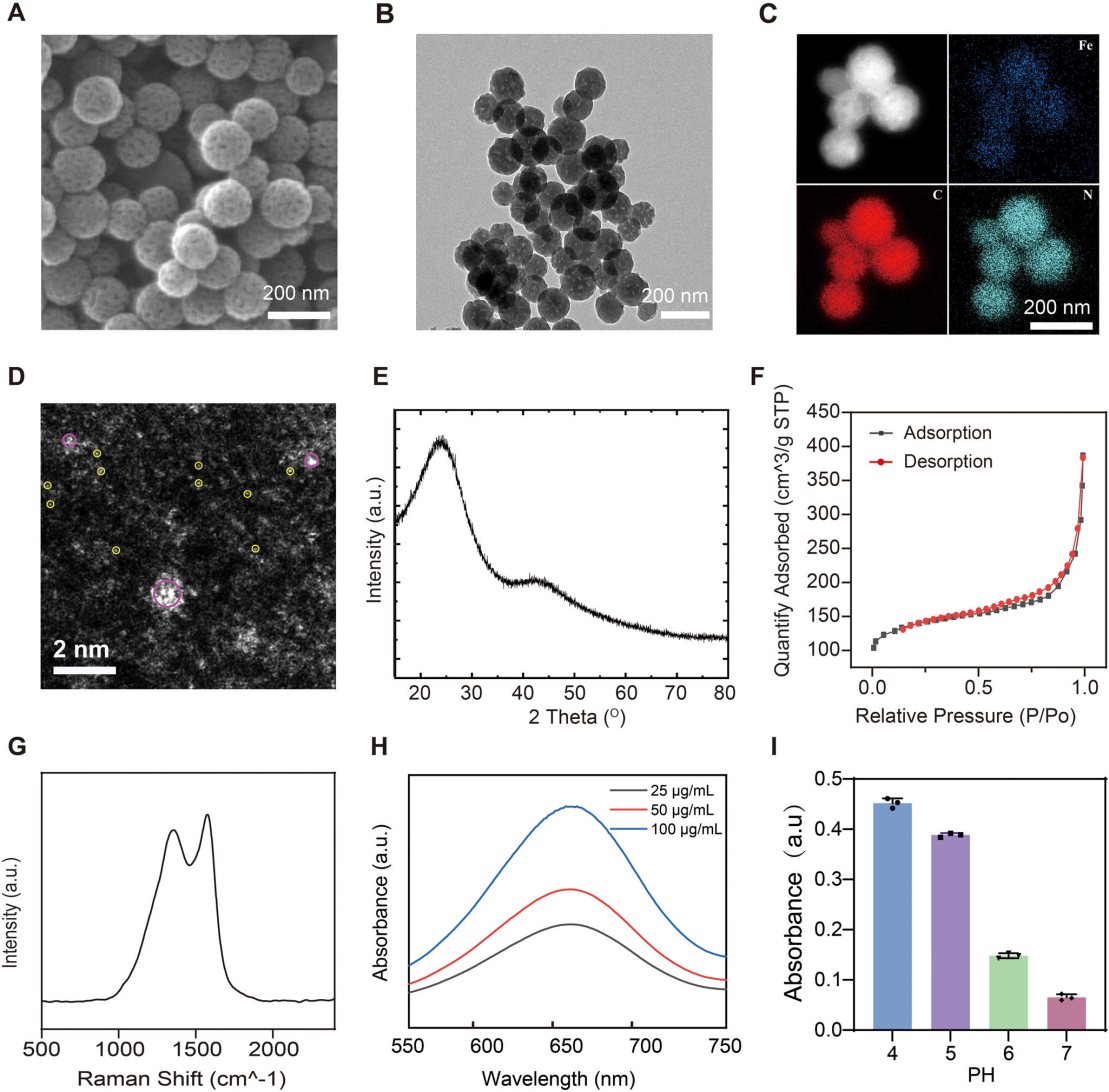
Characterization of the Linco@Fe‐zyme. (A) SEM and (B) TEM images of the Fe‐zyme; (C) EDS mapping of the Fe‐zyme showing the distribution of O, C, N, and Fe elements; (D) AC‐HAADF‐STEM images revealing single atomic dispersion of Fe (circled by yellow) and Fe atom clusters (circled by pink) in the Fe‐zyme; (E) XRD of Fe‐zyme; (F) N_2_ absorption of Fe‐zyme; (G) Raman spectra of Fe‐zyme; (H) UV–vis absorption spectra showing oxTMB absorbance at different nanozyme concentrations (pH 6); (I) Measurement of the POD‐like activity of the Fe‐zyme using TMB as a chromogenic substrate under different pH, with absorbance recorded at 652 nm (*n* = 3).

To enable precise delivery of Linco to the tumor site, we designed an Fe─N─C‐based heterogeneous nanozyme composite to encapsulate Linco (denoted as Linco@Fe‐zyme). We first synthesized this heterogeneous nanozyme via an adsorption–calcination strategy, in which dispersed Fe single atoms (Fe SA) and Fe atomic clusters (Fe AC) were co‐supported on an N‐doped carbon matrix (Fe_S_/Fe_C_). Scanning electron microscopy (SEM) and transmission electron microscopy (TEM) observations revealed that the nanozyme with a spherical morphology with a predominant particle size of approximately 110 nm (Figure 3A,B), which is optimal for prolonged circulation and retention in tumor cells. Dynamic light scattering (DLS) analysis showed that the nanozymes had a predominant particle size distribution around 110 nm (Figure S6A). In addition, the material displayed a zeta potential of around −18 mV. To further assess stability, Fe‐zyme was incubated in PBS at 37°C, and its zeta potential was measured at different time points, followed by TEM imaging. Both zeta potential (Figure S6B) and TEM (Figure S6C) results showed no noticeable changes over time, indicating good stability of Fe‐zyme in PBS. These results demonstrate that the Fe‐zyme possesses favorable colloidal stability and is suitable for subsequent tumor therapy studies. Energy‐Dispersive X‐ray Spectroscopy (EDS) mapping images confirmed the uniform distribution of C, O, N, and Fe elements across the nanoparticles (Figure 3C). Using Aberration‐corrected High‐angle Annular Dark‐field Scanning Transmission Electron Microscopy (AC‐HAADF‐STEM, sub‐angstrom resolution), we observed discrete bright spots in Fe_S_/Fe_C_ that correspond to isolated Fe single atoms and Fe atom clusters (highlighted by pink and yellow circles, respectively), indicating the coexistence of two types of Fe active centers (Figure 3D). In addition, X‐ray photoelectron spectroscopy (XPS) was performed to probe the surface chemical states of Fe‐zyme. The XPS survey spectrum (Figure S6D) shows the presence of C, N, and O elements. The high‐resolution Fe 2p spectrum (Figure S6E) further confirms the presence of iron species, where the fitted peaks indicate positively charged Fe without an obvious Fe⁰ signal near ∼706.7 eV. This may be attributed to the relatively low iron content, which makes Fe⁰ difficult to detect by XPS; however, the AC‐HAADF‐STEM results could reveal the existence of Fe clusters [47, 48].

Furthermore, powder X‐ray diffraction (XRD) showed two main reflections at 25.6° and 43.5° (Figure 3E), assignable to the (002) and (100) planes of carbon, with no discernible diffraction features for iron species across the entire pattern. Although SEM and TEM images provided preliminary evidence of mesoporosity, we performed Brunauer‐Emmett‐Teller (BET) surface area analysis to quantitatively assess the pore characteristics (Figure 3F). The resulting N_2_ adsorption‐desorption isotherms further corroborated the mesoporous nature, with the pore‐size distribution showing a peak pore diameter of ∼13 nm (Figure S6F). This mesoporous structure lays the basis for the subsequent drug loading. The FT‐IR spectrum (Figure S6G) shows characteristic broad peaks at 3432, 1561, and 1221 cm^−1^, corresponding to O─H, C═C/C═N, and C─N vibrations, respectively, indicating the formation of a graphitic carbon nitride‐like structure. Moreover, Raman spectroscopy results revealed the presence of amorphous carbon structures on the nanozyme surface (Figure 3G). To evaluate POD activity, 3,3′,5,5′‐tetramethylbenzidine (TMB) was employed as a chromogenic substrate, and a characteristic absorbance peak at 652 nm was observed across all tested nanozyme concentrations (Figure 3H). In addition, we conducted Michaelis–Menten steady‐state kinetic experiments using H_2_O_2_ as the substrate, and the results are provided in Figure S7A,B. The calculated Vmax and Km values were 0.512 µm/s and 2.61 mm, respectively. Electron paramagnetic resonance (EPR) analysis further confirmed the generation of hydroxyl radicals in the system co‐incubated with the nanozyme and H_2_O_2_ (Figure S7C). The reaction exhibited strong pH dependence, with the 652 nm signal markedly enhanced under lower pH conditions (Figure 3I), consistent with more efficient ROS production in the mildly acidic TME.

Recent studies have systematically characterized the catalytic behavior, reaction pathways, and biomedical potential of POD active nanozymes [49], providing an important framework for benchmarking our Fe‐zyme system. The pioneering work by Gao et al. [50] demonstrated that Fe_3_O_4_ nanoparticles possess intrinsic POD‐like activity capable of catalyzing H_2_O_2_ decomposition into hydroxyl radicals, thereby enabling tumor‐selective catalytic therapy. To further verify the source of hydroxyl radicals during the POD‐like catalytic process, we performed an H_2_O_2_ degradation assay, which revealed a significant time‐dependent decrease in H_2_O_2_ concentration in the presence of the nanozyme (Figure S7D), validating that hydroxyl radicals were primarily generated via H_2_O_2_ decomposition.

After co‐incubating Fe‐zyme with tumor cells for 24 h, we measured the levels of lipid peroxidation products, including MDA (Figure S8A) and 4‐HNE (Figure S8B,D). The results showed that Fe‐zyme markedly increased the production of lipid peroxidation metabolites. Consistently, C11‐BODIPY fluorescence analysis demonstrated substantial accumulation of oxidized lipid species in the Fe‐zyme–treated group (Figure S8C,E,F). Furthermore, when N‐acetylcysteine (NAC) was added to scavenge ROS in the co‐incubation system, the levels of lipid peroxidation products were significantly reduced. These findings indicate that Fe‐zyme promotes ferroptosis in tumor cells through its strong ROS‐generating capability.

After confirming the POD‐like catalytic activity of Fe‐zyme, we next evaluated its drug‐loading performance. We established a standard calibration curve for lincomycin via HPLC (Figure S9A). Fe‐zyme exhibited an encapsulation efficiency of 81.41% and a drug loading capacity of 6.31% (Figure S9B). Furthermore, we examined the release behavior of Linco@Fe‐zyme in PBS at neutral pH and in an acidic environment (pH 5.6) mimicking the TME. The results showed that Linco@Fe‐zyme released lincomycin much more rapidly under acidic conditions, achieving nearly 80% drug release within approximately 10 h (Figure S9C). To further evaluate the cytotoxicity of Linco@Fe‐zyme and to determine the optimal drug combination ratio that achieves desirable dose response and synergistic effects, we assessed the combined activity of Fe‐zyme and lincomycin at different concentrations. Using a gradient of 1, 5, 10, 15, and 20 µg/mL, we re‐determined their half‐maximal inhibitory concentrations (IC_50_). The IC_50_ values of Fe‐zyme and lincomycin were calculated to be 7.885 and 6.835 µg/mL, respectively (Figure S9D,E). Based on this gradient, we subsequently evaluated the inhibitory effects of various concentration combinations. We found that the combination of 10 µg/mL lincomycin and 10 µg/mL Fe‐zyme achieved approximately 90% inhibition of tumor cell viability in vitro (Figure S9F). Although the inhibition rate showed minor fluctuations at higher concentrations, it consistently remained around 90%. This ratio was considered sufficient to exert strong cytotoxic effects on tumor cells. Using the Bliss independence model (ET = 1 − (1 − EA) × (1 − EB)), the theoretical inhibition rate was calculated to be 93.17%, while the observed inhibition rate reached 94.44%, exceeding the predicted value and indicating a synergistic interaction between the two agents. Accordingly, the working concentration of Linco@Fe‐zyme was set at 10 µg/mL for subsequent experiments. Collectively, these results confirm the successful synthesis of an iron‐based nanozyme with excellent POD‐like catalytic activity and mesoporous structure, promising the potential as a multifunctional drug delivery platform for gastrointestinal tumor therapy.

### 2.4 In Vitro Evaluation of Ferroptosis Induction and Antitumor Efficacy by Linco@Fe‐zyme

**FIGURE 4:**
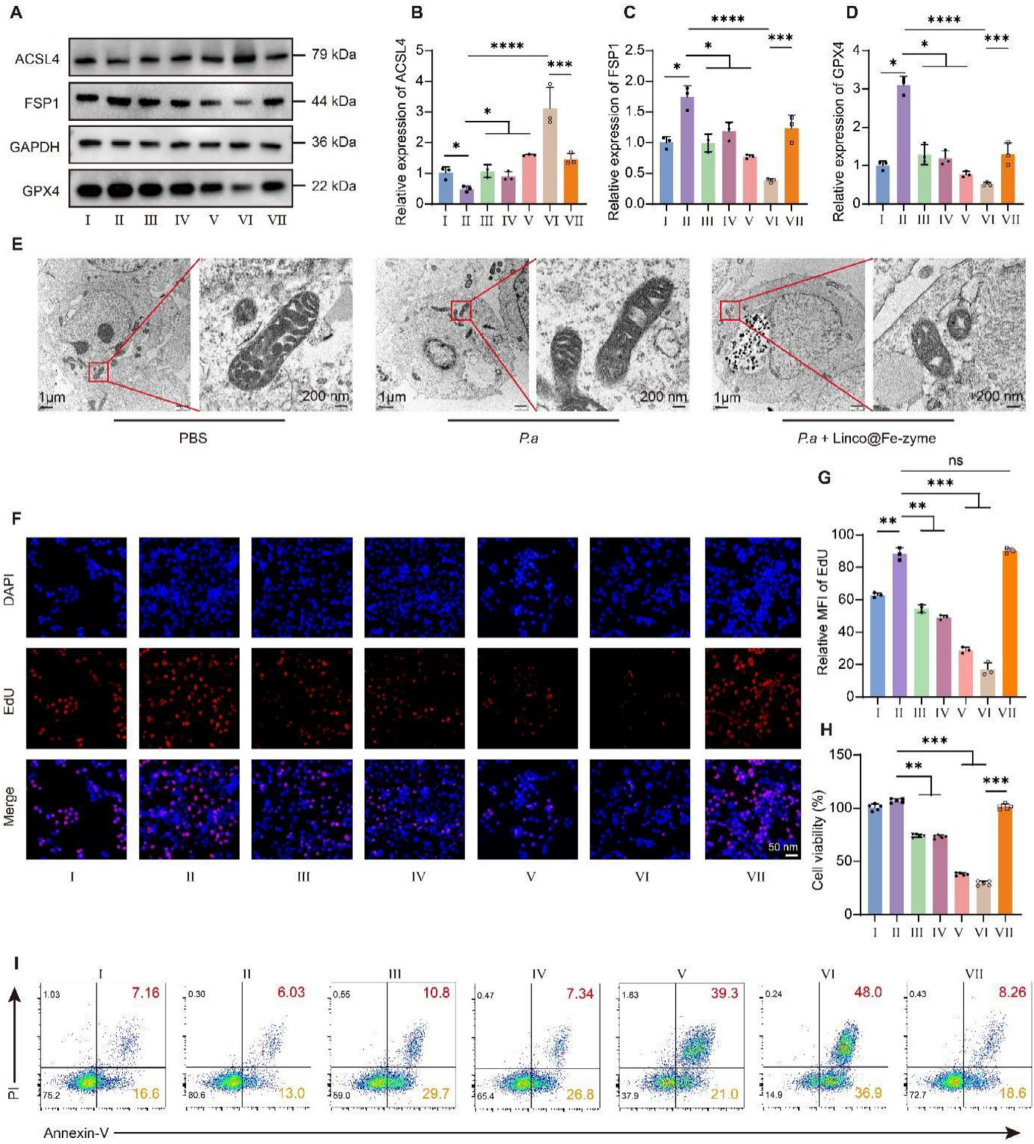
Linco@Fe‐zyme promotes ferroptosis of tumor cells to achieve tumor‐killing effect. (A) Western blot analysis of ferroptosis‐related protein expression across seven treatment groups. (B–D) qPCR results validating ferroptosis induction in CRC cells across the same treatment groups. (E) TEM analysis of LoVo cell mitochondria from PBS, *P. anaerobius*, and Linco@Fe‐zyme groups, revealing mitochondrial shrinkage in the Linco@Fe‐zyme group, scale bar: 1 µm, the scale bar of the right picture = 200 nm. (F) EdU assay indicating reduced proliferation in Linco@Fe‐zyme‐treated LoVo cells, scale bar = 50 µm. (G) Relative MFI of EdU in different treatment groups. (H) CCK‐8 assay assessing cell viability, showing maximal cytotoxicity in the Linco@Fe‐zyme group. (I) Flow cytometry for apoptosis detection, showing increased early and late apoptosis in the Linco@Fe‐zyme group. I:PBS, II:*P.a*, III: *P.a*+Linco, IV: *P.a* + Fe‐zyme, V:*P.a* + Linco + Fe‐zyme, VI: *P.a* + Linco@Fe‐zyme, VII: *P.a* + Linco@Fe‐zyme + Fer‐1 *p < 0.05, **p < 0.01, ***p < 0.001, ****p < 0.0001.

Following the successful synthesis of Linco@Fe‐zyme, we first validated its antibacterial capacity. Antimicrobial testing confirmed that Linco@Fe‐zyme exhibited superior bactericidal activities when compared to free lincomycin (Figure S10). To demonstrate that our nanozyme‐based delivery system provides superior efficacy compared with simple co‐administration of free drugs, we included additional treatment groups in the subsequent experiments: free Fe‐zyme, free lincomycin, and the combination of free Fe‐zyme + free lincomycin. After 48 h of treatment, ferroptosis‐associated protein expression was analyzed. Western blot results showed that *P. anaerobius* treatment suppressed ferroptosis, whereas Linco@Fe‐zyme markedly upregulated ferroptosis‐related markers. (Figure 4A and Figure S11A–C). Consistent with this, qPCR analysis confirmed enhanced ferroptosis signatures in the Linco@Fe‐zyme group (Figure 4B–D). Given that mitochondrial shrinkage is a hallmark of ferroptosis, TEM was employed. Mitochondrial cristae collapse and outer membrane condensation were clearly observed in the Linco@Fe‐zyme treated LoVo cells, while PBS and *P. anaerobius* treated groups showed no significant morphological changes (Figure 4E). The activation of ferroptosis and the destruction of mitochondrial function can lead to the death of LoVo cells [51]. Therefore, we further explored the killing ability of Linco@Fe‐zyme to tumor cells. 5‐ethynyl‐2'‐deoxyuridine (EdU) incorporation assays showed a marked reduction in DNA synthesis, indicating impaired proliferative capacity in Linco@Fe‐zyme treatment group. Notably, the ferroptosis inhibitor restored the proliferative ability of the cells (Figure 4F,G). CCK‐8 assays demonstrated that Linco@Fe‐zyme treatment led to the highest LoVo cell death rate among all groups (Figure 4H). Additionally, Annexin‐V/PI flow cytometry revealed a significant increase in early (right lower quadrant) and late (right upper quadrant) apoptotic populations following Linco@Fe‐zyme treatment, suggesting concurrent induction of apoptosis (Figure 4I). These results support that Linco@Fe‐zyme effectively enhances ferroptotic signaling and exerts potent antitumor effects in LoVo cells. Collectively, these data indicate that the Fe‐zyme drug delivery system possesses a stronger ferroptosis‐activating capability and antitumor effect than the simple co‐administration of free drugs.

### 2.5 In Vitro Validation of Dendritic Cell Maturation Induced by Linco@Fe‐zyme

With the aggravation of ferroptosis in LoVo cells, the lysed tumor cells release many intracellular contents, which serve as DAMPs to promote DC maturation [52]. This process ultimately activates cytotoxic T lymphocytes (CTLs) and enhances antitumor immune responses [53]. Among the most prominent features of this process are the extracellular release of high mobility group box 1 (HMGB1), the upregulation and cytoplasmic relocalization of calreticulin (CRT), and the leakage of ATP [54].

**FIGURE 5:**
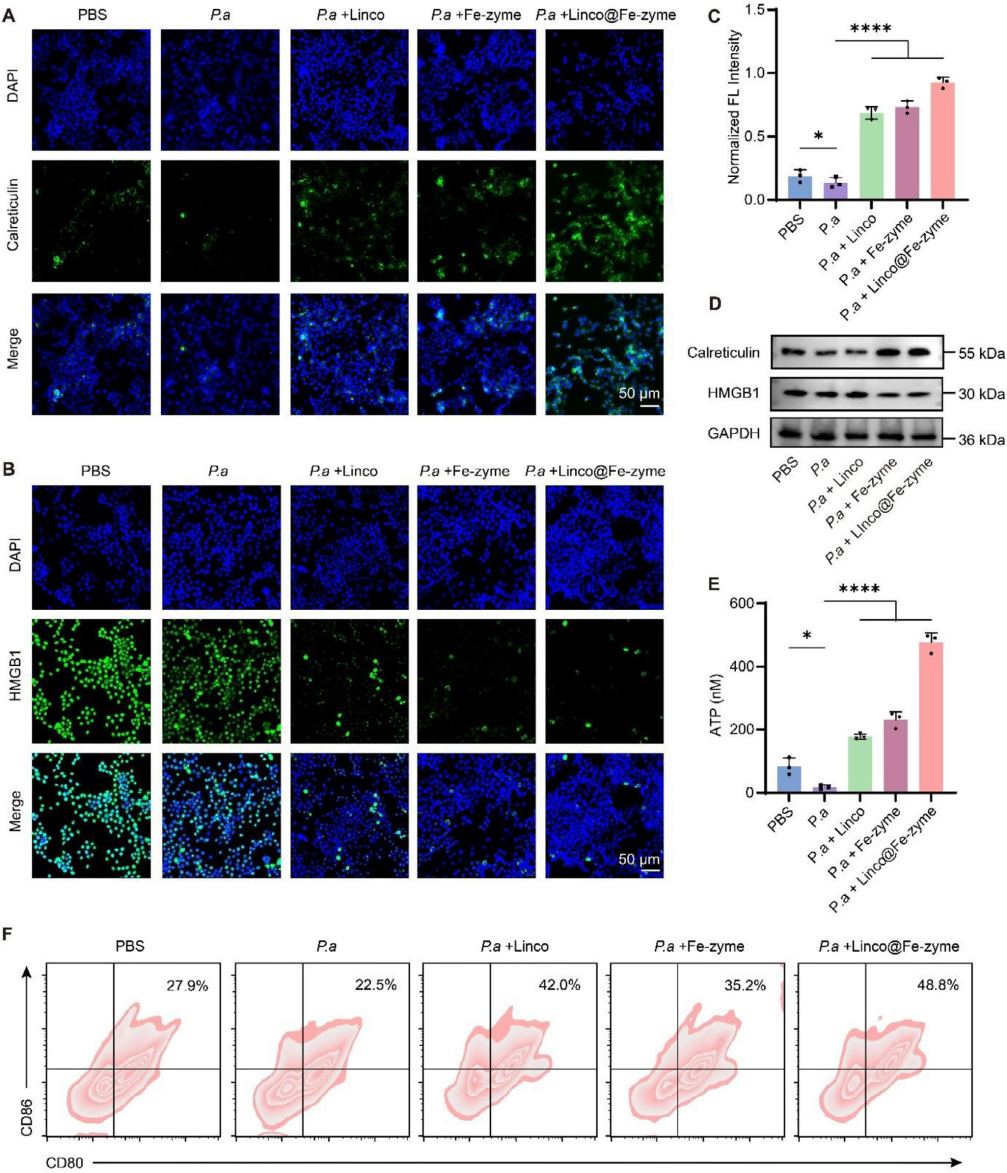
Linco@Fe‐zyme enhances tumor cell ferroptosis‐mediated release of tumor‐associated antigens to potentiate antitumor immunity. (A) Immunofluorescence imaging of intracellular CRT in LoVo cells following different treatments. Scale bar: 50 µm. (B) Immunofluorescence imaging of intracellular HMGB1 in LoVo cells subjected to different treatments. Scale bar: 50 µm. (C) Quantitative analysis of CRT fluorescence intensity in LoVo cells. (D) Western blot analysis of intracellular HMGB1 and CRT levels in LoVo cells after various treatments. (E) Quantification of extracellular ATP released from LoVo cells after various treatments. (F) Flow cytometric analysis of CD80 and CD86 expression in BMDCs isolated from C57BL/6 mice and co‐cultured with LoVo cells subjected to different treatments. *p < 0.05, ****p < 0.0001.

To investigate whether ferroptosis in LoVo cells triggers DAMP release, we performed fluorescence imaging to assess HMGB1 and CRT levels in LoVo cells. As shown in Figure 5A, the cells with Linco@Fe‐zyme treatment exhibited stronger CRT fluorescence signals than those in other groups. Concurrently, a reduction in intracellular HMGB1 suggested enhanced extracellular release (Figure 5B), while Figure 5C and Figure S12 present the quantitative analysis of CRT and HMGB1 fluorescence intensity. These findings were further corroborated by Western blot (Figure 5D). Moreover, the Linco@Fe‐zyme group demonstrated significantly higher extracellular ATP release compared with other treatments (Figure 5E). These data confirm that Linco@Fe‐zyme induced ferroptosis in LoVo cells generates abundant DAMPs, thereby providing tumor‐associated antigens to potentiate antitumor immunity. Subsequently, to verify whether DAMPs released from ferroptotic tumor cells could indeed promote DC maturation, bone marrow–derived dendritic cells (BMDCs) were isolated from mice and co‐cultured with tumor cells subjected to different treatments for 24 h. BMDCs were then harvested and analyzed by flow cytometry to evaluate their maturation status by investigating the CD11c, CD80, and CD86 expression. The results showed that BMDCs in the Linco@Fe‐zyme group displayed markedly higher proportions of CD80^+^ and CD86^+^ cells (Figure 5F), indicating a greater population of mature DCs. Together, these findings demonstrate that tumor cells treated with Linco@Fe‐zyme release substantial amounts of tumor‐associated antigens, which effectively activate DCs and enhance antitumor immune responses.

### 2.6 In Vivo Validation of Ferroptosis Induction and Antitumor Efficacy of Linco@Fe‐zyme

**FIGURE 6:**
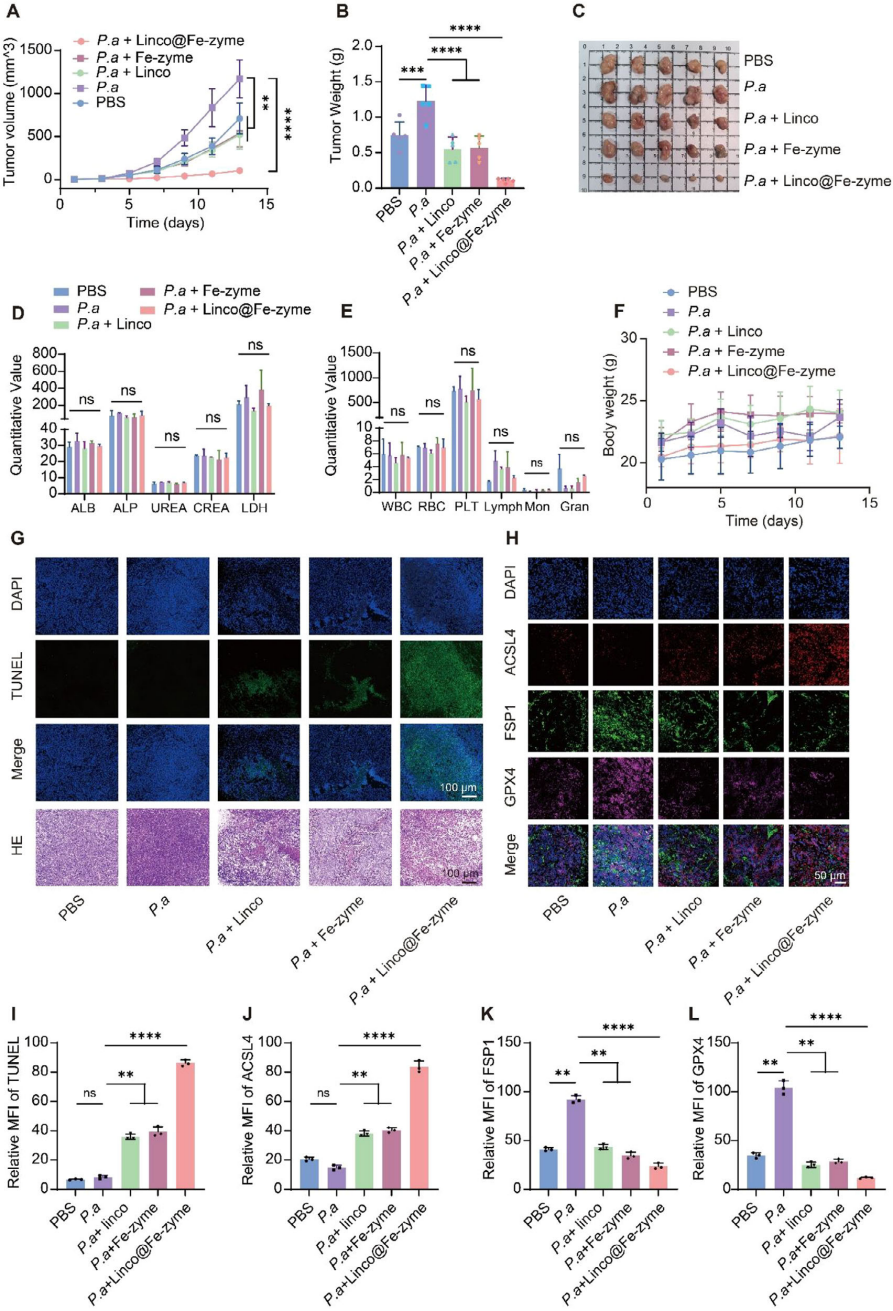
In vivo evaluation of therapeutic effect of Linco@Fe‐zyme. (A) Tumor growth curves for five treatment groups. (B) Tumor weight comparisons among groups. (C) Representative tumor photographs. (D,E) Blood biochemical and hematological parameters, indicating no systemic toxicity. (F) Body weight changes during treatment. (G) TUNEL and H&E staining of tumor tissues; Linco@Fe‐zyme induced the most extensive tumor cell death. Scale bar: 100 µm. (H) Immunofluorescence staining of GPX4, FSP1, and ACSL4 in tumor tissues, indicating ferroptosis modulation. Scale bar: 50 µm. (I) Quantification of TUNEL staining across different treatment groups. (J–L) Quantitative fluorescence analysis of ACSL4, FSP1, and GPX4 expression in the various treatment groups. *p < 0.05, **p < 0.01, ***p < 0.001, ****p < 0.0001, ns = no significant difference.

Following the in vitro validation, the therapeutic efficacy of Linco@Fe‐zyme was evaluated in tumor‐bearing C57BL/6 mice. As shown in Figure 6A, the tumor volume with Linco@Fe‐zyme treatment was smaller than that in other groups, while the *P. anaerobius*‐treated group showed the most aggressive tumor growth. Both tumor weights (Figure 6B) and representative tumor images (Figure 6C) confirmed the superior antitumor performance of Linco@Fe‐zyme. To assess biosafety, after 14 days of treatment, three mice from each group were sacrificed, and blood samples and organ tissues were collected for detection of relevant indicators. No significant differences were observed in hematological or biochemical parameters across groups (Figure 6D,E). Furthermore, histological examination (H&E staining) of major organs (intestine, heart, liver, spleen, lung, and kidney) revealed no apparent tissue toxicity (Figure S13), and body weights remained stable throughout the treatment period (Figure 6F). Collectively, these findings demonstrate that Linco@Fe‐zyme exhibits excellent biosafety with no significant in vivo toxicity. Further, tumors were harvested and subjected to H&E and TUNEL staining. Linco@Fe‐zyme treatment resulted in the highest levels of tumor cell death (Figure 6G,I). Furthermore, multiplex immunofluorescence staining of ferroptosis markers showed elevated ACSL4 and reduced GPX4 and FSP1 expression in the group with Linco@Fe‐zyme treatment, indicating potent ferroptosis activation. In contrast, tumors from *P. anaerobius*‐treated mice exhibited strong ferroptosis resistance (Figure 6H,J‐L). These data confirm that Linco@Fe‐zyme effectively promotes ferroptosis and exerts robust antitumor activity in vivo.

**FIGURE 7:**
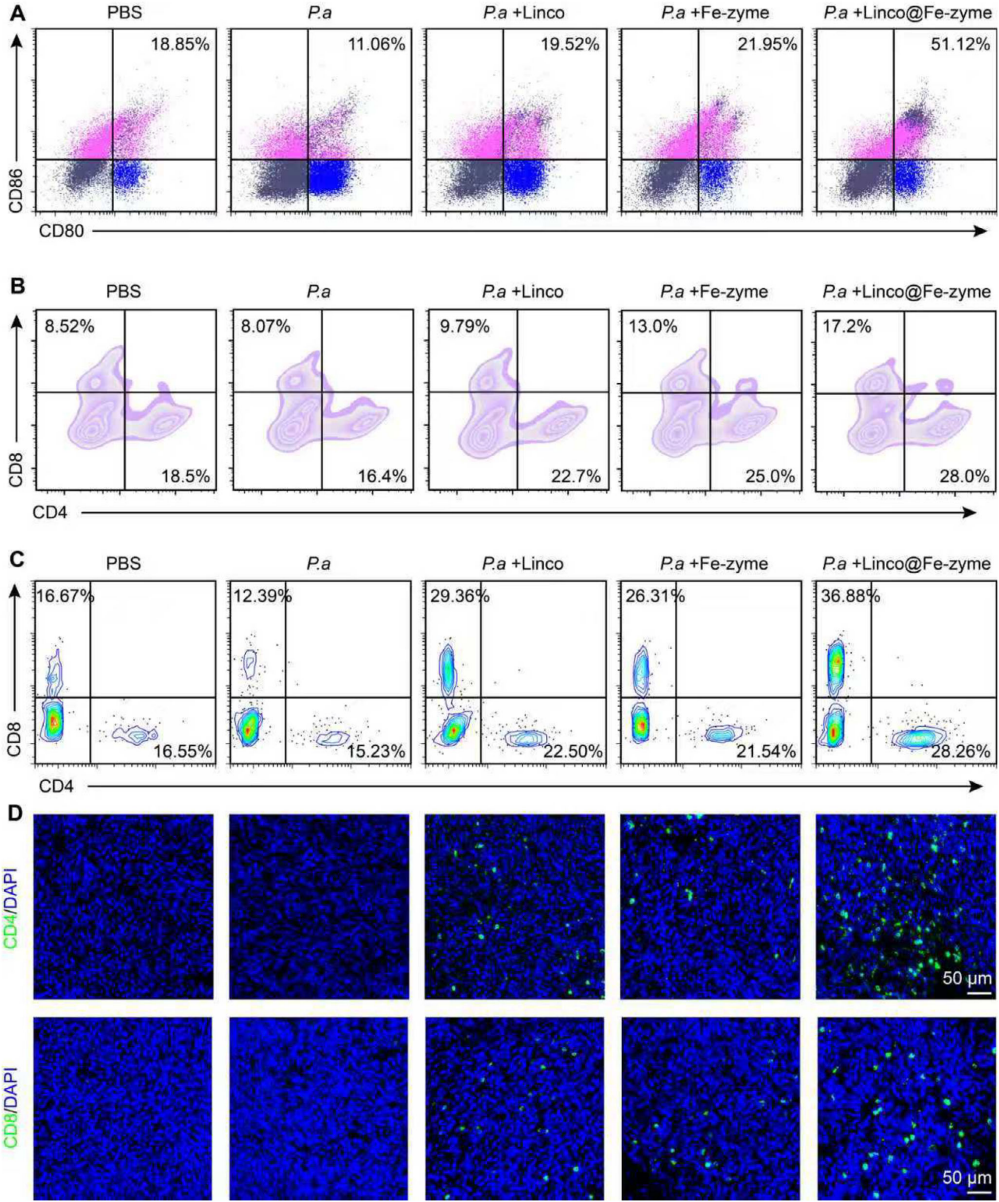
In vivo antitumor immunity assessment of different treatment group. (A) Flow cytometry analysis of mature DC (CD11c^+^CD80^+^CD86^+^) in mice spleens after different treatments. (B) Flow cytometry analysis of memory T cells (CD3^+^CD4^+^) or effector T cells (CD3^+^CD8^+^) in mice spleens after different treatments. (C) Flow cytometry analysis of memory T cells (CD3^+^CD4^+^) or effector T cells (CD3^+^CD8^+^) in tumor after different treatments. (D) Immunofluorescence images of tumors after different treatments stained with DAPI and anti‐CD4 antibody or anti‐CD8 antibody, respectively, scale bar: 50 µm.

To evaluate the activation of antitumor immunity in vivo, we assessed the maturation of DCs and the T cell activation in mice from different treatment groups. Specifically, single cell suspensions were prepared from spleens collected from each group, and the proportion of mature DCs (CD11c^+^, CD80^+^, CD86^+^) was analyzed by flow cytometry (Figure 7A). The Linco@Fe‐zyme treated group exhibited the highest percentage of CD80^+^ and CD86^+^ mature DCs, consistent with our in vitro BMDC results. We next examined splenic T cell populations (Figure 7B). In the Linco@Fe‐zyme group, both CD3^+^CD4^+^ memory T cells and CD3^+^CD8^+^ effector T cells were markedly increased compared with other groups. Furthermore, analysis of tumor tissues revealed significantly greater infiltration of both memory and effector T cells in the Linco@Fe‐zyme group (Figure 7C). Immunofluorescence imaging of tumor sections further corroborated these findings (Figure 7D). These results demonstrate that Linco@Fe‐zyme treatment promotes DC maturation and activates T cells, thereby enhancing antitumor immune responses in vivo.

### 2.7 Microbial and Metabolomic Analysis Reveals Mechanism of Ferroptosis Reversal by Linco@Fe‐zyme

**FIGURE 8:**
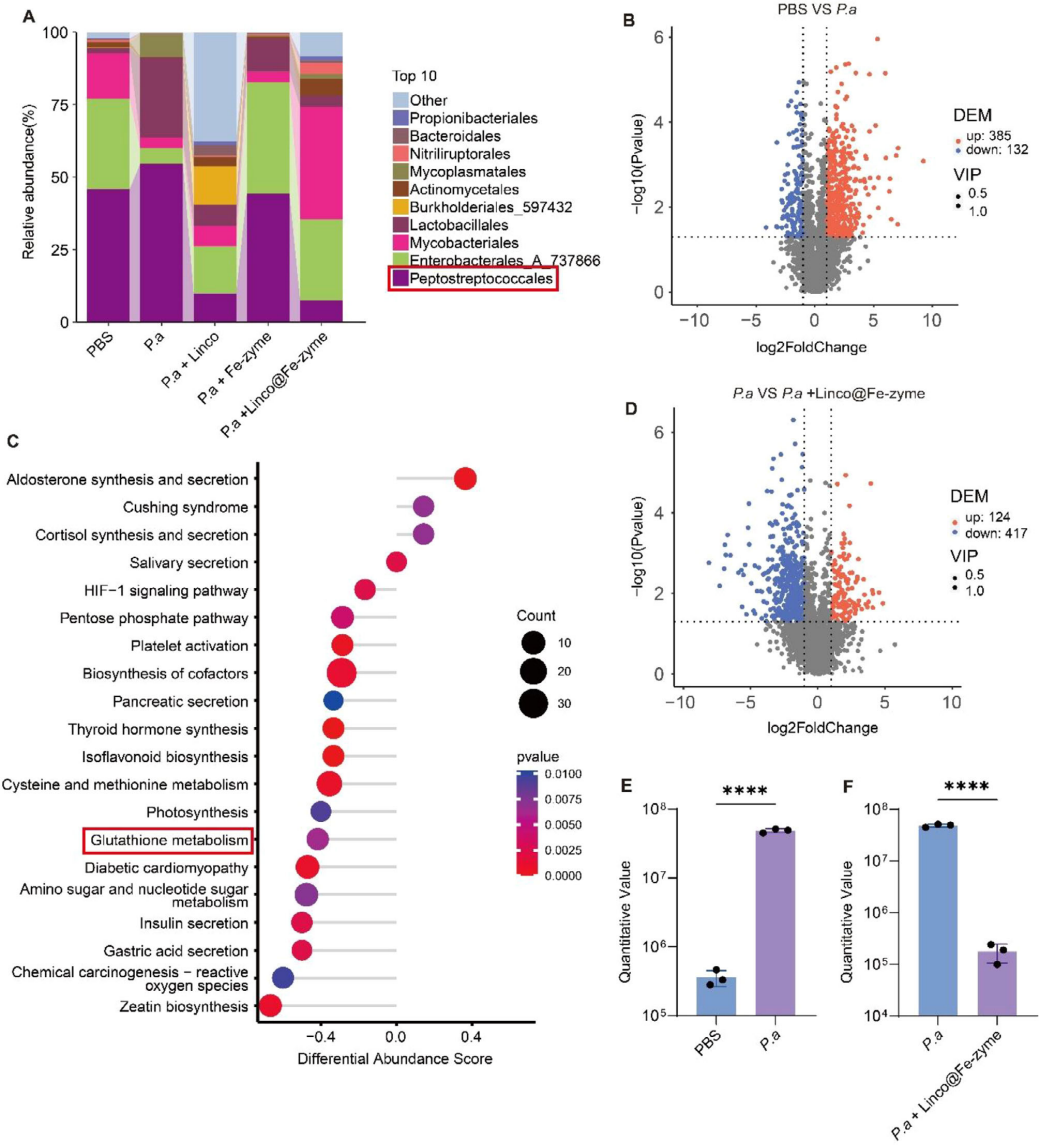
Microbiota and metabolite analysis. (A) 16S sequencing showing reduced *P. anaerobius* abundance in Linco@Fe‐zyme‐treated tumors. (B) Volcano plot comparing microbial metabolites between PBS and *P. anaerobius* groups. (C) KEGG enrichment bubble plot indicating glutamine metabolism involvement. (D) Volcano plot showing reduced metabolites in Linco@Fe‐zyme group. (E,F) Quantification of IDA levels across groups, confirming that Linco@Fe‐zyme decreases IDA abundance. ****p < 0.0001.

To investigate whether Linco@Fe‐zyme eliminates intratumoral *P. anaerobius* and thereby reverses CRC ferroptosis resistance, fresh‐frozen tumor tissues were subjected to 16S rRNA sequencing and metabolomics analysis. The results showed that the overall microbial community structure in the Linco@Fe‐zyme treatment group shifted markedly toward a composition resembling that of the healthy PBS group. Notably, the relative abundance of *P. anaerobius* was dramatically reduced after Linco@Fe‐zyme treatment (Figure 8A). This reduction was substantially greater than that achieved by free Linco or Fe‐zyme alone, highlighting the enhanced antibacterial efficacy of the targeted delivery system. Beyond suppressing *P. anaerobius*, Linco@Fe‐zyme treatment also restored the balance of several beneficial commensal taxa, including *Bacteroidales*, *Lactobacillales*, and other dominant orders typically disrupted during dysbiosis. As a result, the microbial community shifted from a pathogenic, tumor‐associated profile toward a more balanced and homeostasis‐supporting ecosystem. This compositional recovery suggests that Linco@Fe‐zyme not only eradicates harmful bacteria but also mitigates microbiota dysregulation induced by *P. anaerobius*, potentially contributing to reduced inflammation, improved epithelial integrity, and a more favorable metabolic environment within the TME.

Volcano plots revealed that *P. anaerobius* exposure increased the levels of multiple microbial metabolites (Figure 8B), while Linco@Fe‐zyme treatment significantly reduced these metabolites (Figure 8D). KEGG pathway enrichment analysis demonstrated that the affected metabolites were predominantly involved in glutamine metabolism (Figure 8C), a pathway known to regulate ferroptosis. Further analysis revealed that IDA was significantly elevated in the *P. anaerobius* group but markedly reduced after Linco@Fe‐zyme treatment (Figure 8E,F). These findings suggest that Linco@Fe‐zyme effectively eradicates ferroptosis‐antagonizing bacteria and reduces IDA levels, thus restoring ferroptosis sensitivity in CRC.

## 3 Conclusion

In summary, we identified *P. anaerobius* as a key microbial driver of ferroptosis resistance via IDA‐mediated suppression. Its high abundance in CRC tissues and strong correlation with ferroptosis inhibition offer new insights into the microbiota–metabolism–cell death axis and highlights microbial interference as a major obstacle in CRC therapy. To address this challenge, we constructed Linco@Fe‐zyme, a lincomycin‐loaded, POD‐active nanozyme capable of targeted delivery which exhibits excellent biosafety, efficiently eliminates intratumoral *P. anaerobius*, reduces IDA production, and restores ferroptosis sensitivity in CRC. Additionally, the POD‐like catalytic activity of the nanozyme further enhances ferroptosis through ROS generation. The synergy of *P. anaerobius* elimination and ROS‐mediated lipid peroxidation markedly accelerates tumor cell ferroptosis. Subsequently, ferroptotic tumor cells release abundant tumor‐associated antigens, triggering the release of DAMPs and inducing a robust ICD response. Compared to conventional antibiotics or single‐mode ferroptosis inducers, Linco@Fe‐zyme provides enhanced specificity and therapeutic synergy, positioning it as a versatile tool for microbiota‐ and metabolism‐targeted CRC therapy.

## 4 Experimental Section

### 4.1 Clinical Specimen Collection

The CRC samples were taken from patients who underwent surgical resection. A total of 25 patients with pathologically diagnosed CRC were enrolled in this study. The Institutional Review Board of the Union Hospital of Tongji Medical College, Huazhong University of Science and Technology, conducted a review and approved the research protocol and its ethical considerations (2024‐0830).

### 4.2 Cell Culture

LoVo cells (human colorectal adenocarcinoma, RRID: CVCL_0399) were purchased from American Type Culture Collection (ATCC) in 2023, and MC38 cells (murine colon adenocarcinoma, RRID: CVCL_B288) were obtained from the China Center for Type Culture Collection (CCTCC) in 2023. All cell lines were authenticated by short tandem repeat (STR) profiling and confirmed to be free of bacterial, fungal, and mycoplasma contamination prior to experimentation. These two colorectal cancer cell lines, originating from human and murine species respectively, were selected to validate our conclusions across species. Cells were cultured in DMEM supplemented with 10% fetal bovine serum (Gibco) at 37°C in a 5% CO_2_ incubator (Healforce). Cells were digested using trypsin‐EDTA solution (0.25%) for further experiments.

### 4.3 Gut Microbiota Screening

CRC tumor specimens were collected from clinical patients and submitted to Bioyi Biotechnology Co., Ltd., Wuhan, China for 16S rRNA microbial diversity and metabolomics analysis.

### 4.4 Bacterial Inoculation

*Peptostreptococcus anaerobius* (B81243) was activated and cultured on agar plates under anaerobic conditions using anaerobic gas packs. Colonies were collected with sterile inoculating loops to prepare bacterial suspensions, which were mixed with 20% glycerol and administered to nude mice via oral gavage at a concentration of 1 × 10⁹ CFU/mL, twice a week for 3 weeks.

### 4.5 Minimum Inhibitory Concentration (MIC) Assay

Bacterial strains were streaked on agar plates and incubated anaerobically at 37°C (80% N_2_ + 10% CO_2_ + 10% H_2_) overnight until visible colonies formed. Fresh plate cultures were washed with sterile PBS, and the suspension was adjusted to an OD₆₀₀ of 0.6. The bacterial suspension was then inoculated into Todd–Hewitt Broth (Hopebio, HB0311‐3) at 0.1% (v/v). For MIC testing, 200 µL of the inoculated broth was added to the first well of a 96‐well plate, and 100 µL was added to each of the remaining wells. A 25.6 mg/mL stock solution of each antibiotic (Tinidazole: RHAWN, R006362‐5g, ≥98%; Tetracycline: Yuanye, S17051‐5g; Levofloxacin: RHAWN, R01069‐5g; Penicillin: RHAWN, R002182‐5g; Cephalosporin: RHAWN, R014304‐1g; Azithromycin: RHAWN, R014324‐1g; Chloramphenicol: RHAWN, R007448‐5g; Lincomycin: MCE, HY‐117660) was prepared, and 20 µL of each stock was added to the first well. A two‐fold serial dilution was performed by transferring 100 µL from the first well to the second, mixing thoroughly, and continuing this process down to the 11th well, after which 100 µL was discarded from the final well. Medium alone served as the negative control. Plates were incubated anaerobically at 37°C for 48 h. Bacterial growth was assessed by measuring OD₆_2_₀ in a microplate reader and by visually examining the wells for turbidity or pellet formation.

### 4.6 Minimum Bactericidal Concentration (MBC) Assay

Antibiotics were prepared at concentrations corresponding to 1/4 MIC, 1/2 MIC, MIC, and 2× MIC. Each concentration was mixed with the bacterial suspension as described in the MIC assay, and the mixtures were plated onto agar plates. After anaerobic incubation at 37°C for 48 h, colony‐forming units (CFUs) were counted. The MBC was defined as the lowest antibiotic concentration that resulted in a ≥99.9% reduction in bacterial viability compared with the initial inoculum, corresponding to fewer than 10 CFUs on the plate.

### 4.7 Time‐Kill Curve Assay

The bacterial suspension was adjusted to an OD₆₀₀ of 0.6 and then diluted to approximately 10⁸ CFU/mL. Ten milliliters of the diluted suspension was transferred into screw‐cap tubes, followed by the addition of antibiotics at the desired concentrations. Cultures were incubated anaerobically at 37°C for the indicated time points. At each time point, samples were serially diluted tenfold in PBS, and 10 µL of the undiluted and diluted suspensions were spotted onto TH agar plates. After anaerobic incubation at 37°C for 24 h, colony numbers were recorded to evaluate the bactericidal kinetics.

### 4.8 Antibiotic Screening Experiments

For the antibiotic screening, *P. anaerobius*. was grown on blood agar plates. Four uniformly sized wells were punched into the agar, and Linco and Linco@Fe‐zyme were added to the wells at a concentration of 10 µg/mL (20 µL per well). After 24 h of incubation, the diameter of the inhibition zones was measured with a ruler to evaluate the antimicrobial activity.

### 4.9 Co‐Culture of Bacterial Supernatant and Tumor Cells

*P. anaerobius*. was cultured as previously described. The culture supernatant was collected by centrifugation at 5000 rpm for 10 min, followed by filtration through a 0.22 µm membrane to remove bacteria. The sterile supernatant was then co‐incubated with tumor cells.

### 4.10 Synthesis of Linco@Fe‐zyme

First, the mesoporous carbon spheres precursors were synthesized. In a typical procedure, 0.5 g of DA was dissolved in 100 mL of a water/ethanol mixture (1:1 with *v/v*) under stirring till a transparent solution was formed. Subsequently, 2.0 mL of concentrated ammonium hydroxide was introduced into the system, and the mixture was stirred continuously for 2 h to initiate polymerization. The resulting polymeric intermediates were isolated by centrifugation, washed with ethanol 8 times, and then subjected to freeze‐drying for 12 h. For Fe‐zyme, 100 mg of dried carbon precursor was ultrasonically dispersed in 15 mL of deionized water. An aqueous solution of FeCl_3_·6H_2_O (37 mm) was slowly added under stirring, and the dispersion was maintained under continuous agitation at room temperature for 6 h to ensure sufficient metal coordination. The obtained intermediates were again collected by centrifugation and freeze‐dried overnight. Then the dried powders were subjected to pyrolysis at 800°C for 3 h under an argon atmosphere, with a temperature ramp rate of 5°C/min.

### 4.11 Characterization of Linco@Fe‐zyme

The structure and morpholog of Fe/FeSAzyme were characterized using field‐emission transmission electron microscopy (FE‐TEM, JEM‐F200). To visualize atomically dispersed Fe species, aberration‐corrected high‐angle annular dark‐field scanning transmission electron microscopy (AC‐HAADF‐STEM) was performed on a JEM‐ARM200F microscope (JEOL, Japan) equipped with a spherical aberration corrector. Nitrogen adsorption–desorption measurements were carried out at 77 K on a Tristar II 3020 surface area analyzer (Micromeritics, USA) to determine the specific surface area. Raman spectroscopy was performed at room temperature using an InVia Raman microscope (Thermo DXR, USA) to investigate the carbon structure. Additionally, ESR spectroscopy, employing 5,5‐dimethyl‐1‐pyrroline N‐oxide (DMPO) as a spin‐trapping agent, was utilized to detect the generation of ·OH during catalytic processes. XPS measurements were performed on an ESCALAB 250Xi instrument using a monochromatic Al Kα source (1486.58 eV) under ultrahigh vacuum. Samples were pressed onto carbon tape, and the binding energies were calibrated to the C 1s peak. FT‐IR spectra were collected at room temperature in the range of 4000–400 cm^−1^ with a resolution of 4 cm^−1^, and each spectrum was averaged over 16 scans with automatic background subtraction.

### 4.12 Peroxides‐Like Steady‐State Kinetic Assays of Fe‐zyme

The steady‐state kinetic assays of Fe‐zyme (50 µg/mL) toward H_2_O_2_ were carried out at 25°C in HAc–NaAc buffer (pH 6.5) using TMB (1 mm, dissolved in ethonal) as the chromogenic substrate. The reaction was monitored at 651 nm on a FlexStation 3 microplate reader at different time points. Kinetic parameters were obtained by fitting the initial reaction rates to the Michaelis–Menten equation.

v=(Vmax∗[S])/(Km+[S])

*v* represents the initial velocity, *V*_max_ is the maximum reaction rate, [S] is the substrate concentration, and *K*_m_ is the Michaelis constant.

### 4.13 Encapsulation Efficiency and Loading Capacity

A series of lincomycin standard solutions at different concentrations was prepared to generate a calibration curve. Lincomycin was then mixed with Fe‐zyme at a predetermined ratio and stirred to allow drug loading. After successful complexation, the mixture was centrifuged and filtered to collect the supernatant containing unencapsulated lincomycin. The concentration of free lincomycin was quantified using the standard curve, and the encapsulation efficiency and loading capacity were calculated according to the following formulas:

$$\begin{array}{ccc} & & \mathrm{Encapsulation}\;\mathrm{efficiency}\;(\mathrm{EE}\%)\\ & & \;=(1-\mathrm{unloaded}\;\mathrm{lincomycin}/\;\mathrm{total}\;\mathrm{lincomycin})\;\times \;100\% \end{array}$$

$$\begin{array}{ccc} & & \mathrm{Loading}\mathrm{capacity}(\mathrm{LC}\%)\\ & & \;=(1-(\mathrm{unloaded}\mathrm{lincomycin}/(\mathrm{total}\mathrm{lincomycin}\\ & & \;+\;\mathrm{total}\;\mathrm{Fe}-\mathrm{zyme}))\times \;100\% \end{array}$$

### 4.14 Drug Release Study

Linco@Fe‐zyme was placed into dialysis bags and immersed in buffer solutions of different pH values. At predetermined time points (1, 2, 4, 6, 8, 10, 12, 16, 20, and 24 h), aliquots of the external buffer were collected to measure the concentration of released lincomycin. The cumulative release profile was calculated based on the measured lincomycin content.

### 4.15 Enzyme Activity of Linco@Fe‐zyme

The POD catalytic activity was evaluated based on the oxidation of 3,3′,5,5′‐tetramethylbenzidine (TMB). In a typical assay, 3.0 mL of acetate buffer solution containing 1 mM TMB and 3 mM H_2_O_2_ was prepared at different nanoenzyme concentration and different pH values (4, 5, 6, 7). After incubating at 25°C for 3 min, the UV‐vis‐NIR absorbance spectra were recorded using a Shimadzu UV‐2550 spectrophotometer (Japan). To assess the H_2_O_2_ consumption, Fe‐zyme was incubated with H_2_O_2_ in 0.01 M HAc‐NaAc buffer (pH 6.0) at 37°C.

### 4.16 Transwell Assays

Cells were cultured to the appropriate confluence and then serum‐starved for 24 h. The next day, cells were digested, counted, and seeded into the upper chamber of transwell inserts in 24‐well plates (200 µl, 5 × 10^4 cells per well). For invasion assays, a layer of Matrigel (60 µL) was applied to the lower surface. Cells were incubated at 37°C in a 5% CO_2_ incubator for 48 h. Non‐migrated cells on the upper surface were removed, and migrated/invaded cells were fixed, stained with crystal violet, air‐dried, and observed under a microscope.

### 4.17 Wound Healing Assays

For wound healing assays, cells were seeded in six‐well plates to confluence. Using a sterile 100 µL pipette tip, four intersecting lines were scratched. Detached cells were washed away with PBS. Cell morphology was recorded under a microscope at 0 h. Plates were then incubated and imaged at 4, 8, 12, and 24 h to assess wound closure.

### 4.18 Cell Proliferation and Cytotoxicity Assays

For proliferation assays, cells were seeded in 96‐well plates at 2000 cells/200 µL per well. After adherence, cells receive various treatments. Each condition had four replicates. CCK‐8 assays were performed on days 1, 2, 3, 4, and absorbance at 450 nm was measured to generate proliferation curves. For cytotoxicity assays, cells were seeded at 10 000 cells per well in 96‐well plates, treated after adherence, and analyzed the next day using absorbance at 450 nm to assess viability. Cell viability was calculated using Equation (1):

(1) $$\mathrm{Cell}\;\mathrm{viability}\;(\%)=\left(A_{a}-A_{b}\right)/\left(A_{c}-A_{b}\right)/\times 100\%$$

where A_a_ represents the absorbance of the sample, A_c_ represents the absorbance of the control, and A_b_ represents the absorbance of the blank.

### 4.19 Protein Extraction and Western Blotting

Proteins were extracted from treated cells or tissues via lysis and sonication, followed by quantification. Samples were subjected to SDS‐PAGE, transferred to PVDF membranes, blocked, and incubated with primary antibodies overnight. After incubation with secondary antibodies the next day, bands were visualized using a Bio‐Rad imaging system. The uncropped full‐length blots used in this study are provided in Figure S14.

### 4.20 RT‐qPCR

RNA was extracted using the Vazyme RNA extraction kit, and concentration was measured with a spectrophotometer. For *P. anaerobius*, RNA extraction was performed by adding preheated (95°C) Vazyme Bacteria RNA Enhancement Reagent during the lysis step, followed by RNA purification according to the standard procedure described above. cDNA was synthesized via reverse transcription. PCR was performed and samples were loaded for amplification and analysis. Primer sequences are listed in Table S1.

### 4.21 Immunohistochemistry and Immunofluorescence Staining

Fresh tissue samples were fixed with 4% paraformaldehyde and dehydrated in ethanol. Paraffin‐embedded sections were stained using primary and secondary antibodies or other dyes for immunohistochemical and immunofluorescent analysis. Stained sections were imaged under a microscope.

### 4.22 Transmission Electron Microscopy of Mitochondria

Cell pellets from different treatment groups were collected and fixed in electron microscopy fixative. After standard processing steps, mitochondrial morphology was observed under transmission electron microscopy.

### 4.23 Immunofluorescence Staining

Cells or tumor tissues from different treatment groups were collected for immunofluorescence staining. Ed staining was performed using a commercial kit (Beyotime, C0075S). BODIPY 581/591 C11 was performed using a commercial kit (Beyotime, S0043S). Ferroptosis‐related markers, ICD‐related markers were detected using primary and secondary antibody staining.

### 4.24 Detection of ICD Markers

After co‐incubating LOVO cells with PBS, *P. anaerobius*, *P. anaerobius* + Linco (10 µg/mL), *P. anaerobius* + Fe‐zyme (10 µg/mL), and *P. anaerobius* + Linco@Fe‐zyme (10 µg/mL) for 12 h, cells from each group were collected. Proteins and RNA were extracted for WB experiments and PCR detection of HMGB1 and CALR expression. The ATP content in each group was measured using an ATP detection kit (S0026, Beyotime, China).

### 4.25 BMDC Isolation and In Vitro BMDC Maturation

BMDCs were extracted from bone marrow of C57BL/6 mice. The co‐incubation experiment of LoVo and BMDC cells was conducted through the Transwell system. Briefly, Lovo cells were treated with *P. anaerobius*, *P. anaerobius* + Linco (10 µg/mL), *P. anaerobius* + Fe‐zyme (10 µg/mL), and *P. anaerobius* + Linco@Fe‐zyme (10 µg/mL) for 12 h. Then, they were transferred to the upper chamber (0.4 µm) of the six‐well plate Transwell system. BMDCs were seeded at the bottom of the six‐well plate and co‐incubated for another 24 h. After that, BMDC cells in the lower chamber were collected for flow cytometry analysis using FITC‐anti‐mouse CD11c, APC‐anti‐mouse CD80, and PE‐anti‐mouse CD86.

### 4.26 Cell Apoptosis Assay

Cells from different treatment groups were stained according to the apoptosis assay kit protocol and analyzed using a flow cytometer (Beyotime, C‐1062M).

### 4.27 Animal Experiments

Six‐week‐old male SPF‐grade nude mice were purchased from Hubei Beiante Biotechnology Co., Ltd. and housed at the Experimental Animal Center of Tongji Medical College, Huazhong University of Science and Technology (IACUC No: 4691). After 1 week of acclimatization, a subcutaneous xenograft mouse model was established by injecting LoVo cells into the right dorsal flank of each mouse (3 × 10⁶ cells/mouse). Tumor volume was calculated using Equation (2):

(2) $$V=\left(\mathrm{length}\times \mathrm{widt}h^{2}\right)/2$$

### 4.28 AOM/DSS‐Induced Orthotopic Colorectal Tumor Model

Six‐week‐old male SPF‐grade C57BL/6 mice were obtained from Hubei Beiante Biotechnology Co., Ltd. and housed at the same facility. After 1 week of acclimatization, mice received a single intraperitoneal injection of azoxymethane (AOM, 10 mg/kg). One week later, regular drinking water was replaced with 2% DSS‐containing distilled water for 1 week, followed by 2 weeks of regular distilled water. This process was repeated three times (9 weeks total). Hematochezia and tumor formation were observed in situ. Mice were euthanized at week 14.

### 4.29 Measurement of Malondialdehyde (MDA) Content

MDA levels were measured using a commercial assay kit (Servicebio, G4302). The working solution was prepared according to the manufacturer's instructions. Cell pellets were lysed using RIPA buffer, and protein concentration was determined using the BCA assay (Servicebio, G2026). Samples were then mixed with the working solution following the kit protocol. Fluorescence intensity was measured using a microplate reader at Em 553 nm / Ex 525 nm, and MDA content was calculated based on the standard curve. The MDA concentration in each sample was determined using the following formula:

$$\begin{array}{ccc} \mathrm{MDA}\;(\mu \mathrm{mol}/\mathrm{gprot}) & = & \left(OD_{\mathrm{test}}-OD_{\mathrm{blank}}\right)/\left(OD_{\mathrm{standard}}-OD_{\mathrm{blank}}\right)\\ & & \times \;\mathrm{Standard}\;\mathrm{concentration} \end{array}$$

(50μmol/L)Proteinconcentrationofthetestsample(gprot/L)

### 4.30 Activation of Systemic Immune Response

To verify the systemic immunity induced by ferroptosis, we collected the lymph nodes and spleens of mice 14 days after treatment. The tissues were extracted into single‐cell suspensions using collagenase, DNAase, and hyaluronidase, and then stained with CD11c, CD80, and CD86 flow cytometry antibodies. The proportion of mature DC cells in different treatment groups was verified and evaluated. To confirm the activation status of T cells in tumor tissues, we collected tumor tissues from different treatment groups 14 days after treatment, extracted single‐cell suspensions, and stained with CD3, CD4, and CD8. Subsequently, we verified it through flow cytometry experiments.

### 4.31 LC–MS/MS Analysis

Tissues were homogenized, and 200 µL of methanol–water extraction solution (2:8, v/v) was added. The mixture was vortexed for 5 min and centrifuged at 13 200 rpm for 6 min. Then, 50 µL of the supernatant was mixed with 150 µL methanol for protein precipitation, followed by a second centrifugation. An aliquot of 80 µL of the resulting supernatant was used for LC–MS/MS analysis.

### 4.32 Statistical Analysis

All data were analyzed and visualized using RStudio 4.4.1 or GraphPad Prism 10.1.2. Results are expressed as mean ± standard deviation. Comparisons between two groups were performed using independent sample *t*‐tests. One‐way or two‐way ANOVA was used for multiple group comparisons. A *p*‐value of < 0.05 was considered statistically significant (*p < 0.05, **p < 0.01, ***p < 0.001, ****p < 0.0001, ns = no significant).

## Author Contributions

Conceptualization was done by Y.C., J.W., K.C., and X.C. Methodology was done by H.W., X. S, P.Q., and J.Z. Investigation was done by Y.C., J.W., H.W., F.F, and P.Q. Visualization was done by S.L., J.Z., X.C., and K.C. Supervision was done by J.L., C.S., J.Z., X.C., K.C. Original draft was written by Y.C., J.W., H.W., and P.Q. Review writing and editing was done by J.Z., X.C., and K.C. Revision was done by Y.C., J.W., X.C., and K.C.

## Conflicts of Interest

The authors declare no conflicts of interest.

## Supporting information




**Supporting File**: advs73534‐sup‐0001‐SuppMat.docx.

## Data Availability

The data that support the findings of this study are available in the supplementary material of this article.
