# Supplementary material for: Targeting Peptostreptococcus anaerobius with an Iron‐Based Nanozyme Reverses Ferroptosis Resistance and Enhances Antitumor Immunity in Colorectal Cancer
Source: Adv Sci (Weinh). 2026 Jan 12;13(35):e16272. doi: 10.1002/advs.202516272 (PMC13292267; doi:10.1002/advs.202516272)
Supplement: Supplementary file 1 — Supporting File: advs73534‐sup‐0001‐SuppMat.docx. [file ADVS-13-e16272-s001.docx]

**Supporting Information**

**Targeting *Peptostreptococcus anaerobius* with an Iron-Based Nanozyme Reverses Ferroptosis Resistance and Enhances Antitumor Immunity in Colorectal Cancer**

Yinghao Cao, Jun Wang, Hanwenchen Wang, Xiang Sun, Fang Fang, Shiying Li, Jianping Liu, Changrong Shi, Pengyuan Qi*, Jianhua Zou*, Xiaoyuan Chen*, Kailin Cai*

Dr. Y. Cao

Department of Digestive Surgical Oncology, Union Hospital, Tongji Medical College, Huazhong University of Science and Technology, Wuhan, 430022, China.

Dr. J. Wang

Department of Thoracic Surgery, Union Hospital, Tongji Medical College, Huazhong University of Science and Technology, Wuhan, Hubei, 430022, China

Hubei Key Laboratory of Biological Targeted Therapy, Union Hospital, Tongji Medical College, Huazhong University of Science and Technology, Wuhan, Hubei, 430022, China

Dr. J. Wang, Dr. H. Wang, Dr. K. Cai

Department of Gastrointestinal Surgery, Union Hospital, Tongji Medical College, Huazhong University of Science and Technology, Wuhan, Hubei 430022, China

E-Mail: [caikailin@hust.edu.cn](mailto:caikailin@hust.edu.cn)

Dr. Y. Cao, Dr. X. Sun, Dr. F. F, Dr. S. L, Dr. J. Liu, Dr. C. S, Dr. J. Zou, Prof. X. Chen

Departments of Diagnostic Radiology, Yong Loo Lin School of Medicine and College of Design and Engineering, National University of Singapore, Singapore, 119074, Singapore

Nanomedicine Translational Research Program, Yong Loo Lin School of Medicine, National University of Singapore, Singapore, 117597

E-Mail: [chen.shawn@nus.edu.sg](mailto:chen.shawn@nus.edu.sg) , [zoujh-93@nus.edu.sg](mailto:zoujh-93@nus.edu.sg)

Dr. Y. Cao, Dr. P. Qi, Prof. X. Chen

Cancer center, Union Hospital, Tongji Medical College, Huazhong University of Science and Technology, Wuhan, Hubei 430022, China

E-Mail: qpyuan@hust.edu.cn

Prof. X. Chen

Department of Chemical and Biomolecular Engineering, College of Design and Engineering, National University of Singapore, Singapore, 117575

Department of Biomedical Engineering, College of Design and Engineering, National University of Singapore, Singapore, 117575

Department of Pharmacy and Pharmaceutical Sciences, Faculty of Science, National University of Singapore, Singapore, 117544

Clinical Imaging Research Centre, Centre for Translational Medicine, Yong Loo Lin School of Medicine, National University of Singapore, Singapore, 117599

Theranostics Center of Excellence (TCE), Yong Loo Lin School of Medicine, National University of Singapore, Singapore, 138667

**Materials**

**Antibody for Western Blot or immunofluorescence.** Primary antibodies against CD4 and CD8 were purchased from Abcam (USA). Primary antibodys against ACSL4, GAPDH, HMGB1, Calreticulin and 4-Hydroxynonenal was purchased from ABclonal (China), Primary antibodys of FSP1, GPX4 was purchased from Proteintech (China).

**Antibody and reagents for flow cytometry.** PE anti-mouse CD45, APC anti-mouse CD11c, FITC anti-mouse CD80, PE/Cyanine7 anti-mouse CD86, FITC anti-mouse CD3, APC anti-mouse CD4, PerCP/Cyanine5.5 anti-mouse CD8α, TruStain FcX™ (anti-mouse CD16/32), Zombie Aqua™ Fixable Viability Kit was purchased from Biolegnd (USA)

**Table S1.** The primer sequences used for qRT-PCR

| **Name** | **Forward-primer (5’-3’)** | **Reverse-primer (5’-3’)** |
| --- | --- | --- |
| ACSL4 (Human) | GCTCTGTCACACACTTCGACTCAC | TTCCCTGGTCCCAAGGCTGTC |
| FSP1 (Human) | CAAGATCAACAGCTCCGCCTACC | CGTCGGCACAGTCACCAATGG |
| GPX4 (Human) | CCCGATACGCTGAGTGTGGTTTG | TCTTCGTTACTCCCTGGCTCCTG |
| GAPDH (Human) | CACCCACTCCTCCACCTTTGAC | GTCCACCACCCTGTTGCTGTAG |
| ACSL4 (Mouse) | TGGCTCATGTGCTGGAACTGAC | CAATCACCCTTGCTTCCCTTCTTG |
| FSP1 (Mouse) | GCAGGAGTAGAGATGGCAGCAG | CGCACACAGGGCAGGAGTTC |
| GPX4 (Mouse) | ATAAGAACGGCTGCGTGGTGAAG | TAGAGATAGCACGGCAGGTCCTTC |
| *P.anaerobius* | CTGATTTGATGCTTGCATTA | AGCCCCGAAGGGAAGGTGTG |


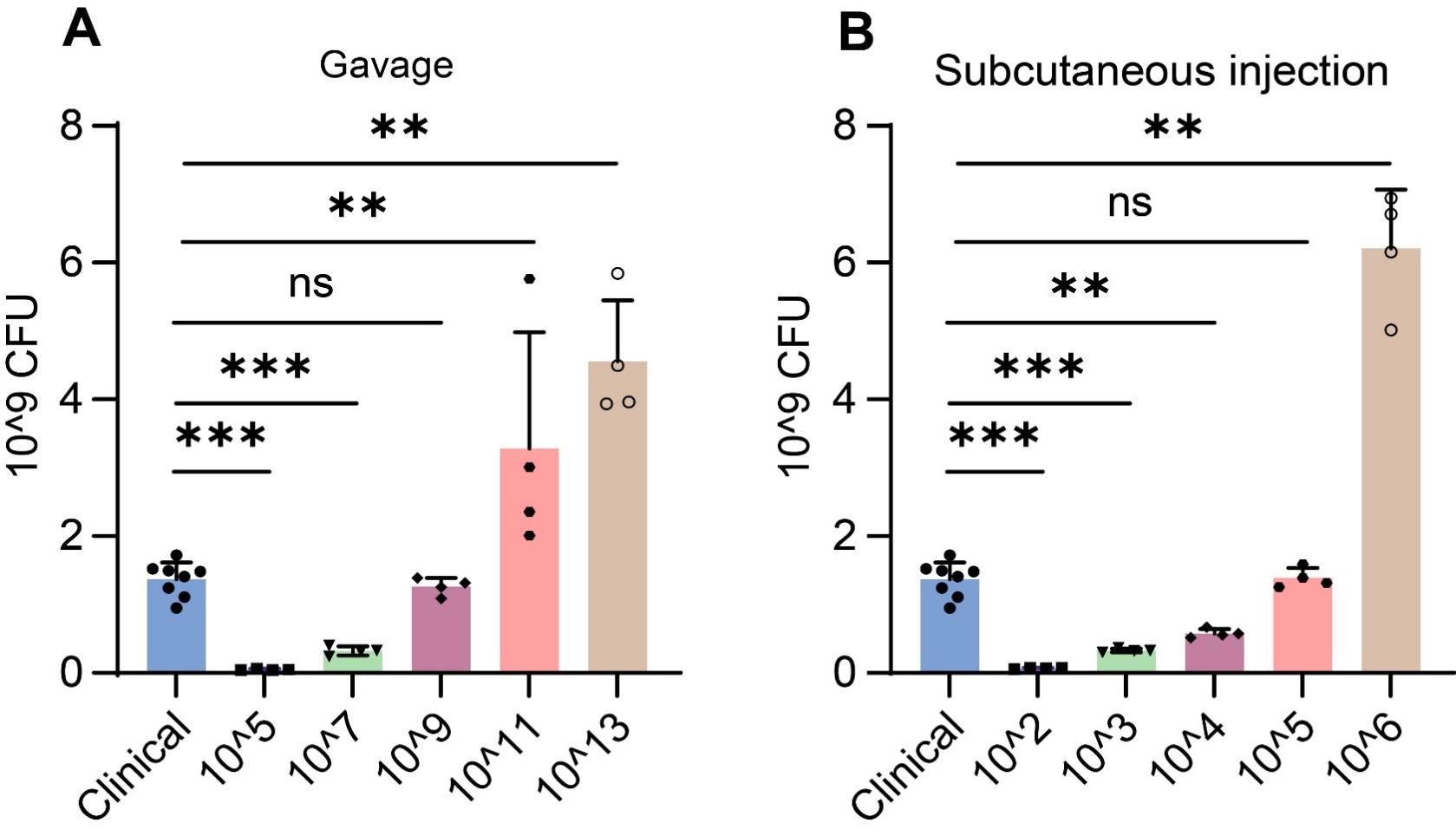


**Figure S1. (A)** PCR analysis of intratumoral P. anaerobius abundance in AOM/DSS orthotopic tumors (n = 3) following treatment with different bacterial doses. Administration of 10^9 CFU bacterial suspension (100 μL), twice per week for two consecutive weeks, resulted in an intratumoral bacterial load comparable to that observed in clinical colorectal tumor tissues (n = 8). **(B)** LC–MS analysis of intratumoral P. anaerobius abundance in subcutaneous tumors (n = 3) after treatment with different bacterial doses. Injection of 10^5 CFU (50 μL per dose), twice per week, achieved clinically comparable bacterial abundance within one week. ** *P* < 0.01, *** *P* < 0.001, ns = no significant difference.


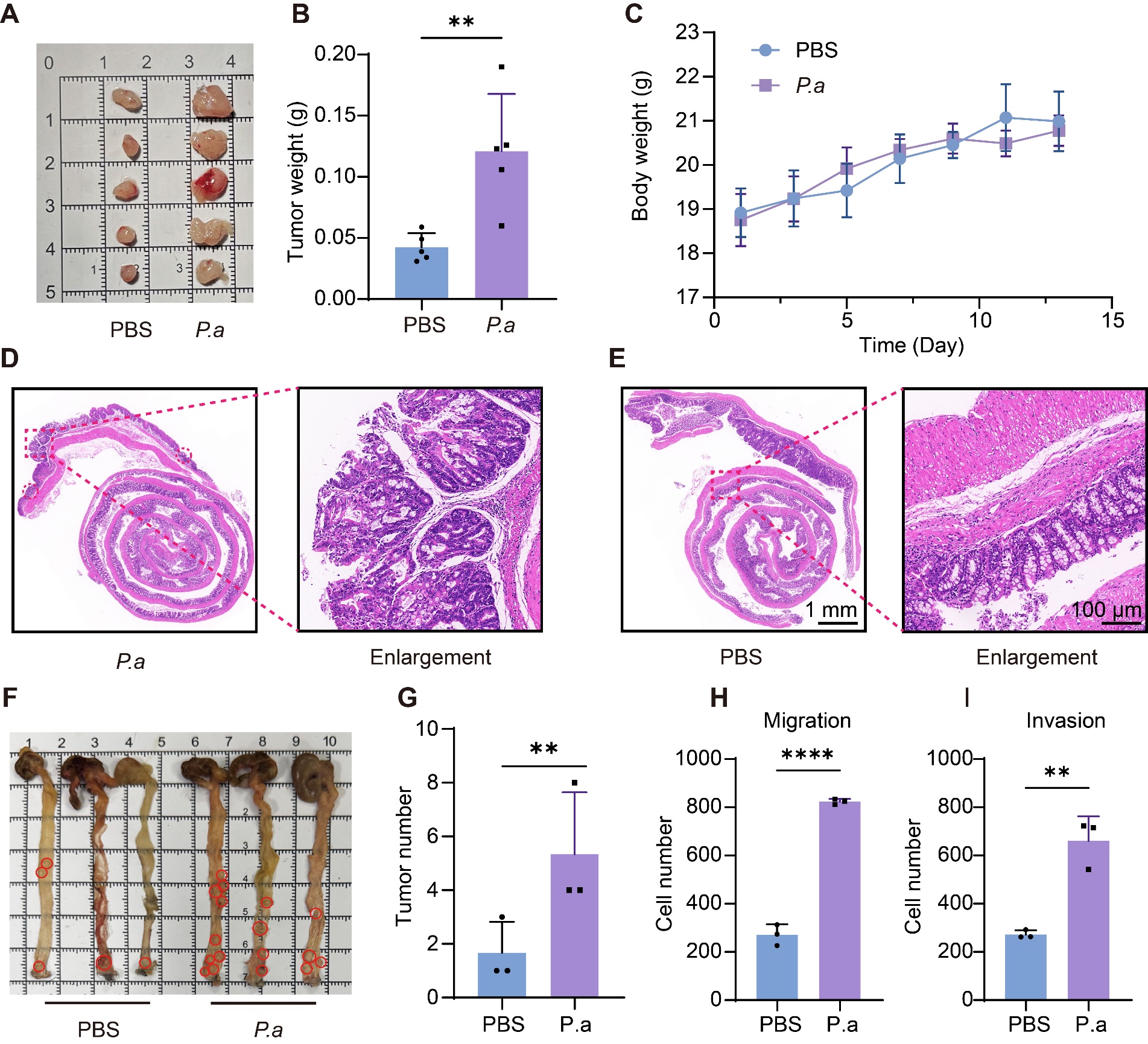


**Figure S2.** **(A)** Gross images of subcutaneous tumors from mice injected with P.anaerobius or PBS; tumors in the P.anaerobius group were visibly larger. **(B)** Comparison of tumor weights between mice stimulated with P.anaerobius and those treated with PBS; **(C)** Body weight changes over two weeks in mice from the P.anaerobius-stimulated group and the PBS-treated group; **(D)** Representative H&E staining image of intestinal tissue from mice treated with P.a, with the dashed circle indicating the region of spontaneous tumor formation; **(E)** Representative H&E staining image of intestinal tissue from PBS-treated mice. **(F, G)** Comparison of the number of in situ tumors formed in the AOM/DSS model under different stimulations, showing a significantly higher number in the P.anaerobius-stimulated group; **(H, I)** Transwell assays comparing the number of tumor cells that migrated and invaded after P.anaerobius or PBS stimulation, indicating that *P. anaerobius* stimulation significantly enhanced tumor cell migration and invasion; ** P < 0.01, **** P < 0.0001.


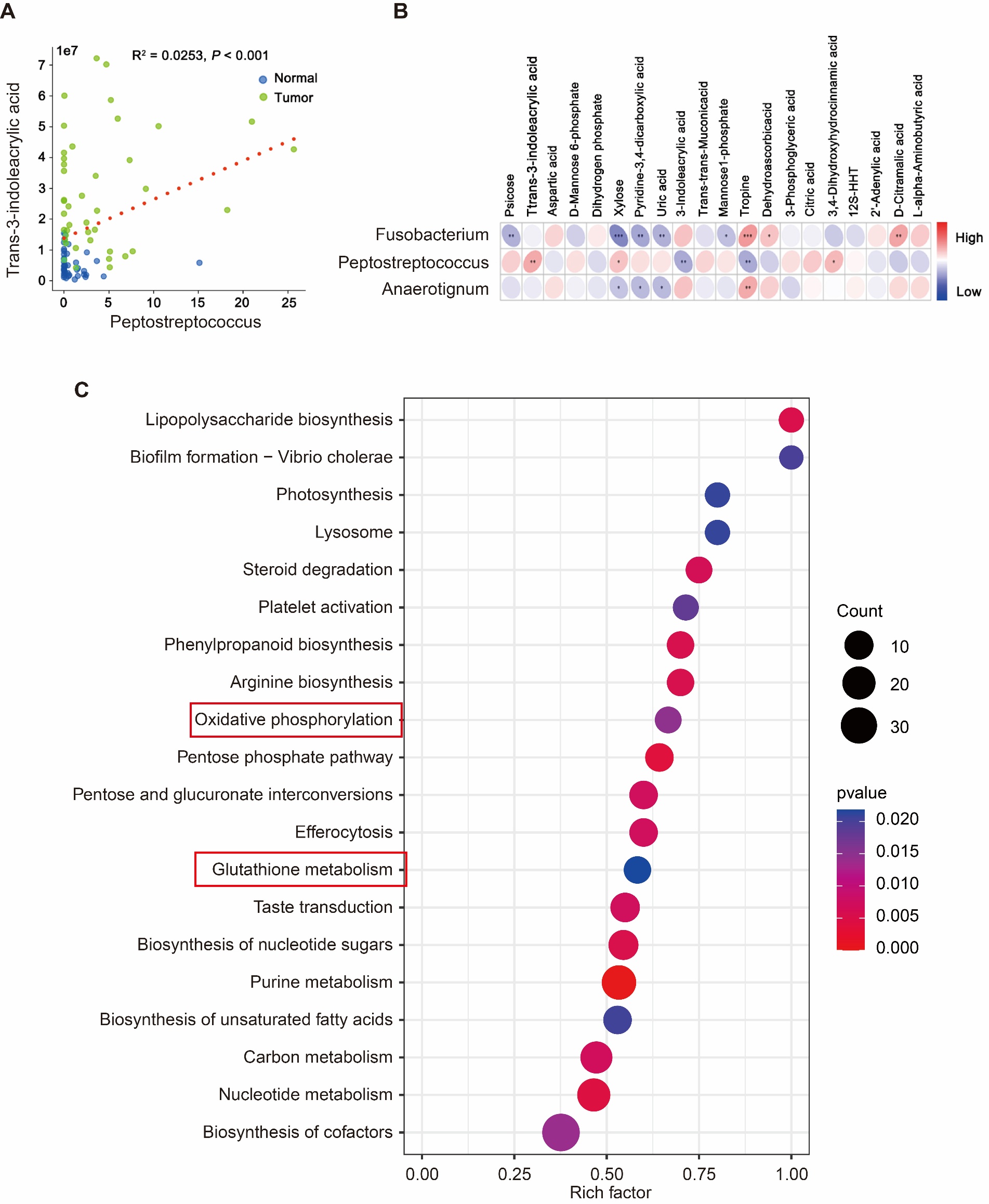


**Figure S3.** **(A)** Quantitative analysis of the *P.anaerobius* metabolite IDA in colorectal cancer tissues and adjacent normal tissues, showing higher levels of IDA in tumor tissues; **(B)** Heatmap of metabolites produced by *P.anaerobius* and two commonly altered bacterial genera identified in 16S rRNA sequencing of colorectal cancer, indicating that *P.anaerobius* produces the highest amount of IDA; **(C)** KEGG pathway enrichment bubble plot of *P.anaerobius* metabolites, showing strong associations with oxidative phosphorylation and glutamine metabolism.


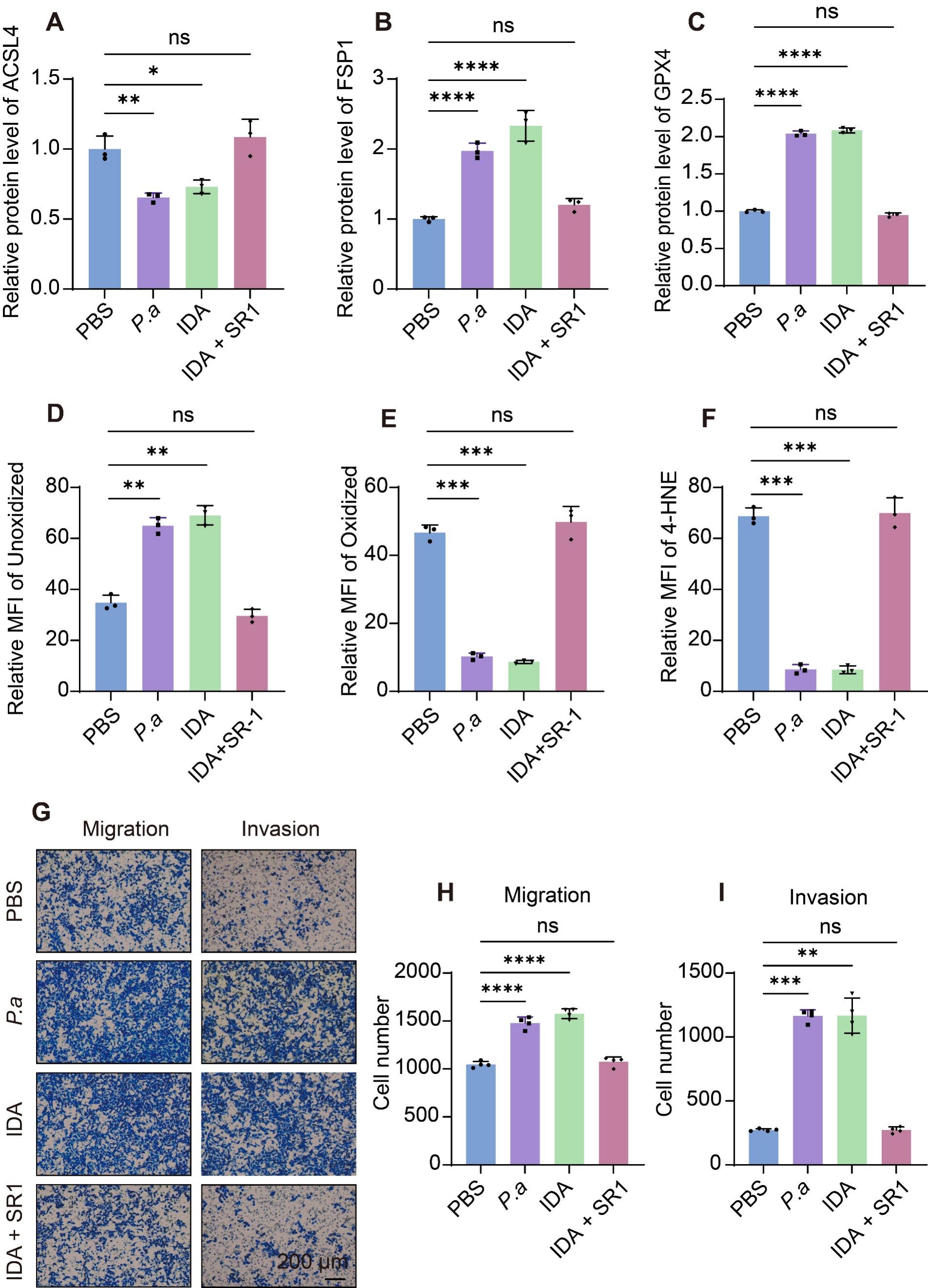


**Figure S4. (A, B, C)** Quantification of Western blot results for three ferroptosis-related markers-ACSL4, FSP1, and GPX4-in tumor cells treated with PBS, *P.anaerobius*, IDA, or IDA+SR1; **(D)** Average fluorescent intensity of non-oxidized red fluorescence detected by C11-BODIPY in different treatment groups. **(E)** Average fluorescent intensity of oxidized green fluorescence detected by C11-BODIPY in different treatment groups. **(F)** Content of lipid peroxidation product 4-HNE in different treatment groups. **(G)** Transwell assay results showing differences in migration and invasion abilities of tumor cells after treatment with PBS, *P.anaerobius*, IDA, or IDA+SR1, magnification = ×200; bar = 200 μm. **(H, I)** Comparison of the number of migrated and invaded tumor cells in Transwell assays among the four groups (PBS, *P.anaerobius*, IDA, and IDA+SR1), indicating the effects of different stimulations. ** P* < 0.05, *** P* < 0.01, **** P* < 0.001, ***** P* < 0.0001, ns = no significant difference.


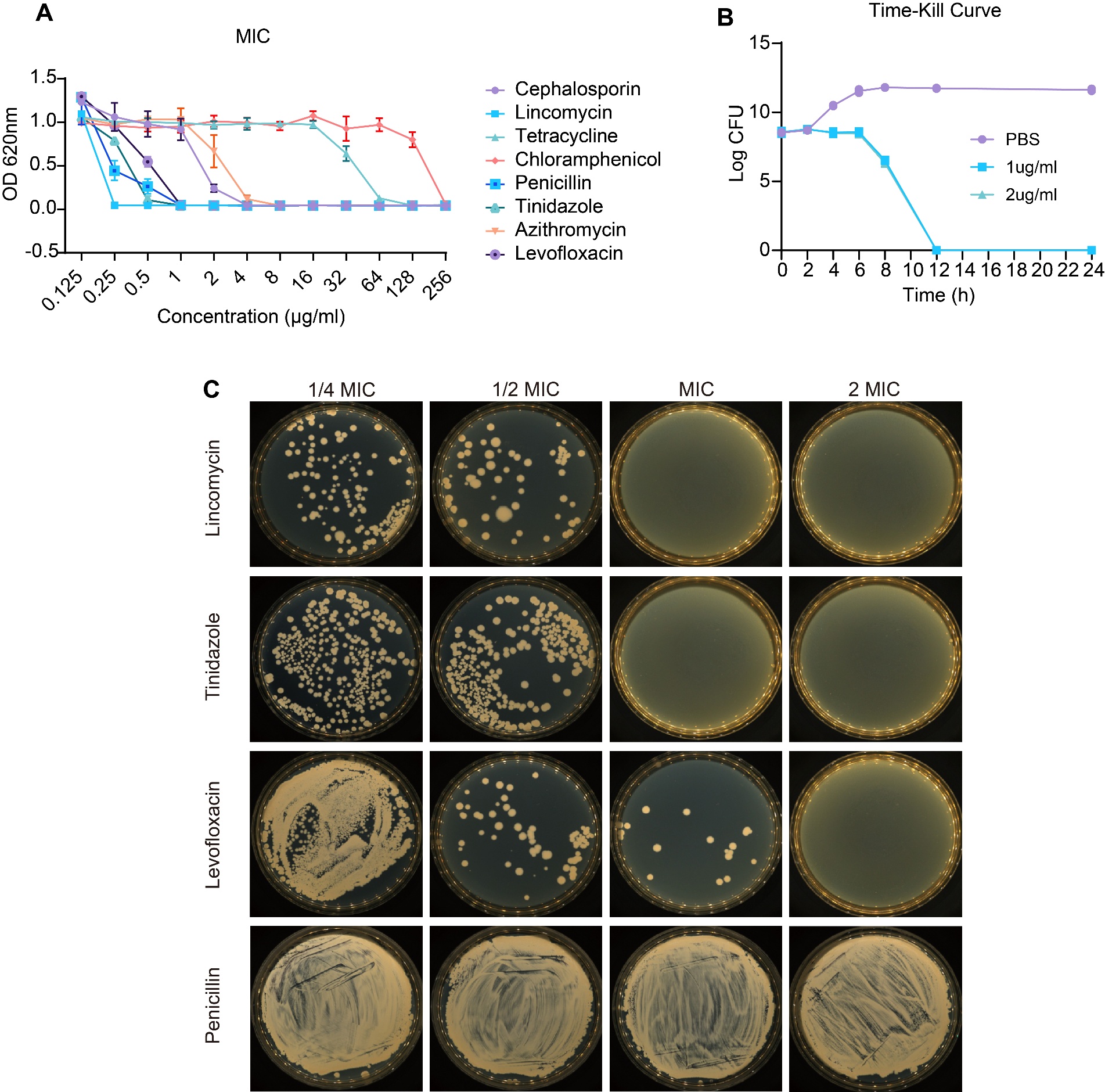


**Figure S5. (A)** MIC curves of different antibiotics, showing that lincomycin reaches its MIC at 0.25 μg/mL. **(B)** Time-kill assays were performed by co-culturing *P.anaerobius* with different concentrations of lincomycin for various time periods, after which the bacteria were collected and replated to assess colony formation. The results showed that at 1 μg/mL lincomycin, almost no viable bacteria remained after 12 hours of co-culture. **(C)** Plate colony formation of *P.anaerobius* under different MIC concentrations of the four antibiotics showed that lincomycin exhibited the strongest bactericidal effect at its MIC level.


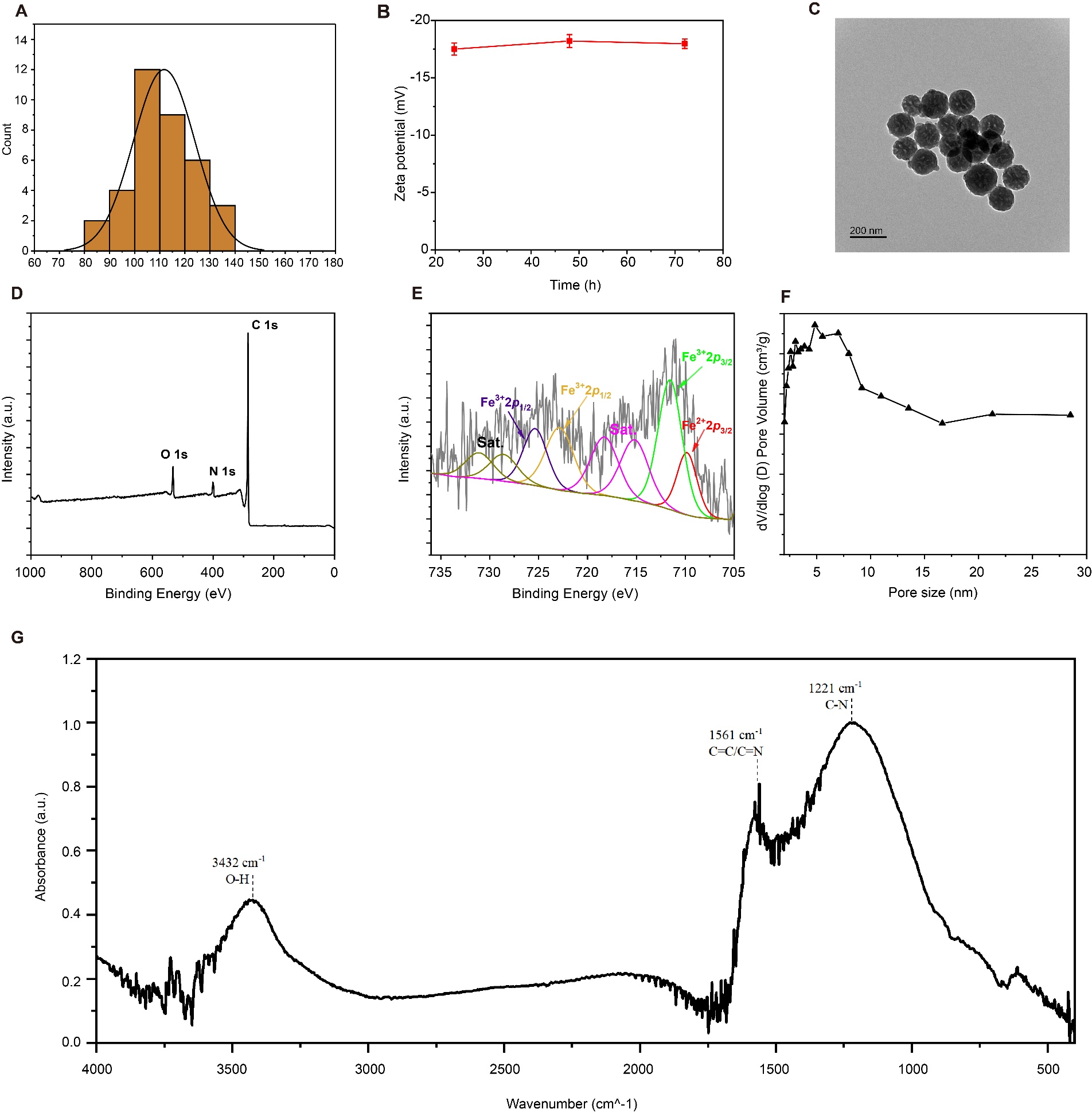


**Figure S6. (A)** Particle size distribution of the Fe-zyme detected by DLS. **(B)** The zeta potential of Fe-zyme measured at different time points. **(C)** The TEM of Fe-zyme after incubating in PBS (pH = 6.5) after 3 days. **(D)** The survey and **(E)** Fe 2p XPS spectra of Fe-zyme. **(F)** Pore size distributions for Fe-zyme. **(G)** The FT-IR spectra of Fe-zyme.


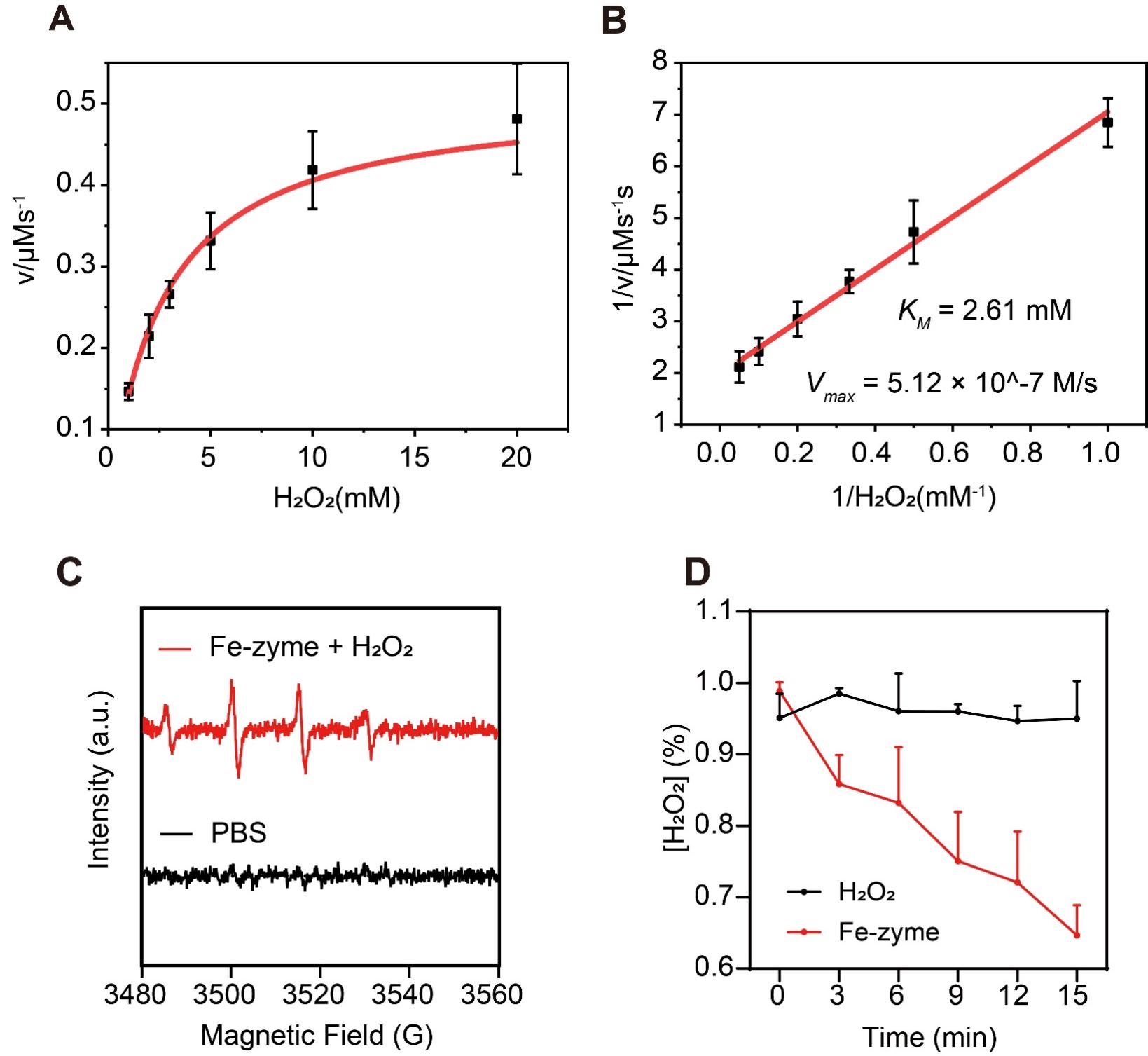


**Figure S7. (A)** Michaelis–Menten kinetic analysis and **(B)** Lineweaver–Burk plot of Fe-zyme (50 mg/mL) using H₂O₂ as the substrate. The pH during measurement was maintained at 6.5. **(C)** Electron spin resonance (ESR) analysis of hydroxyl radical generation after co-incubation of the iron-based nanozyme with H₂O₂; **(D)** Relative UV–vis absorbance spectra of the H₂O₂-Ti(SO₄)₂ solution in the presence of the iron-based nanozyme (pH = 6.0, H₂O₂ concentration = 4.0 mM, Ti(SO₄)₂ concentration = 1%, n = 3).


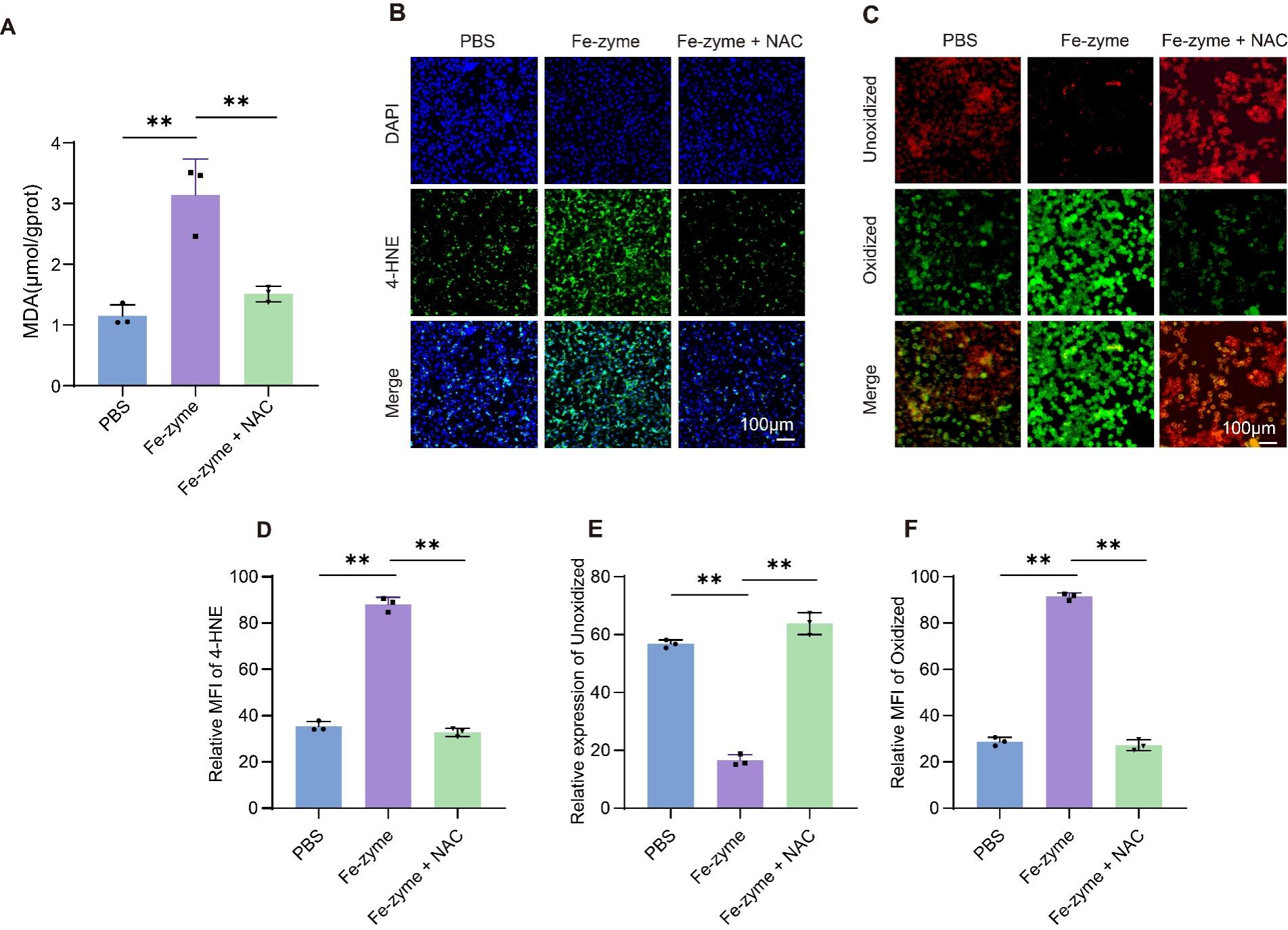


**Figure S8. (A)** Levels of lipid peroxidation product MDA in different treatment groups. **(B)** Levels of lipid peroxidation product 4-HNE in different treatment groups. **(C)** C11-BODIPY fluorescence imaging of lipid peroxidation in different treatment groups; red fluorescence indicates non-oxidized lipids, whereas green fluorescence indicates elevated lipid peroxidation. **(D)** Quantitative analysis of 4-HNE levels in different treatment groups. **(E)** Quantification of non-oxidized red fluorescence detected by the C11-BODIPY probe. **(F)** Quantification of oxidized green fluorescence detected by the C11-BODIPY probe. ***P* < 0.01.


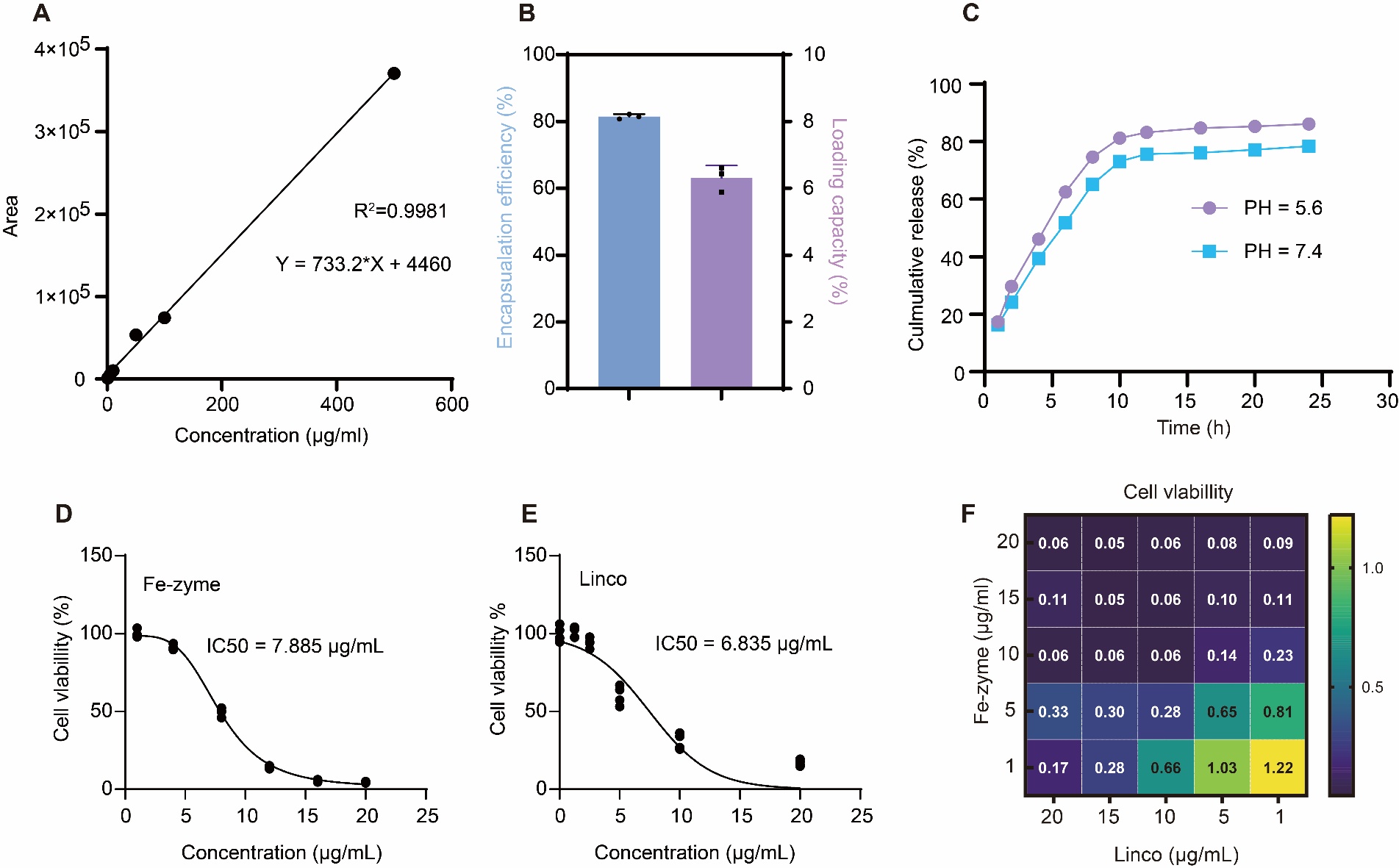


**Figure S9. (A)** Standard calibration curve of lincomycin detected by HPLC. **(B)** Encapsulation efficiency and drug loading capacity of lincomycin on Fe-zyme. **(C)** Time-dependent drug release profile of Linco@Fe-zyme under neutral and acidic pH conditions. **(D)** IC_50_ fitting curve showing the cytotoxic effect of Fe-zyme on tumor cells; **(E)** IC_50_ fitting curve showing the cytotoxic effect of Linco on tumor cells; **(F)** Regarding the combined antitumor effects of Fe-zyme and lincomycin at different concentrations, we found that the combination of 10 µg/mL lincomycin and 10 µg/mL Fe-zyme achieved approximately 90% inhibition of tumor cell viability in vitro.


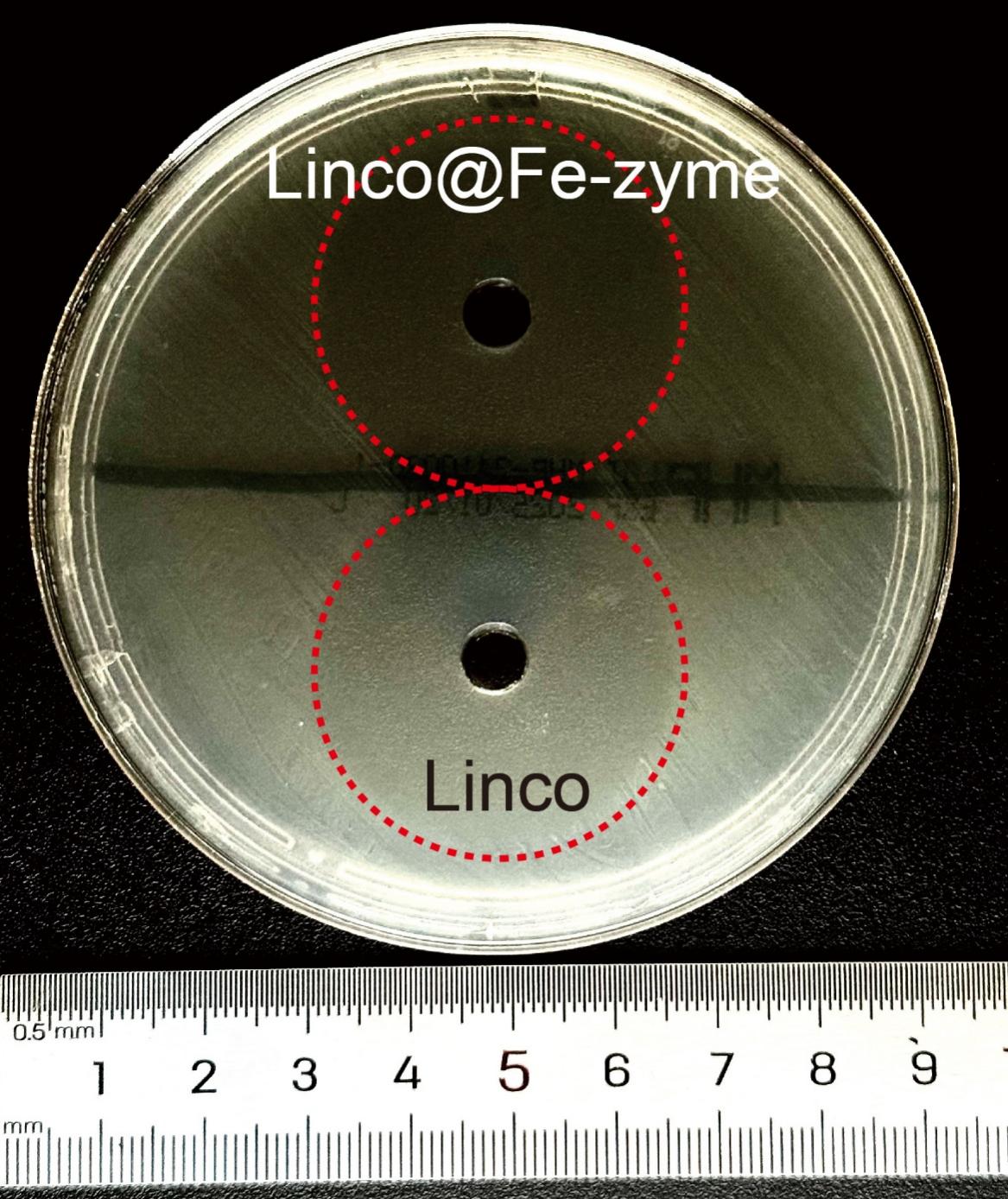


**Figure S10.** Agar diffusion assay comparing the antibacterial activity of Linco@Fe-zyme and Linco against *P.anaerobius*;


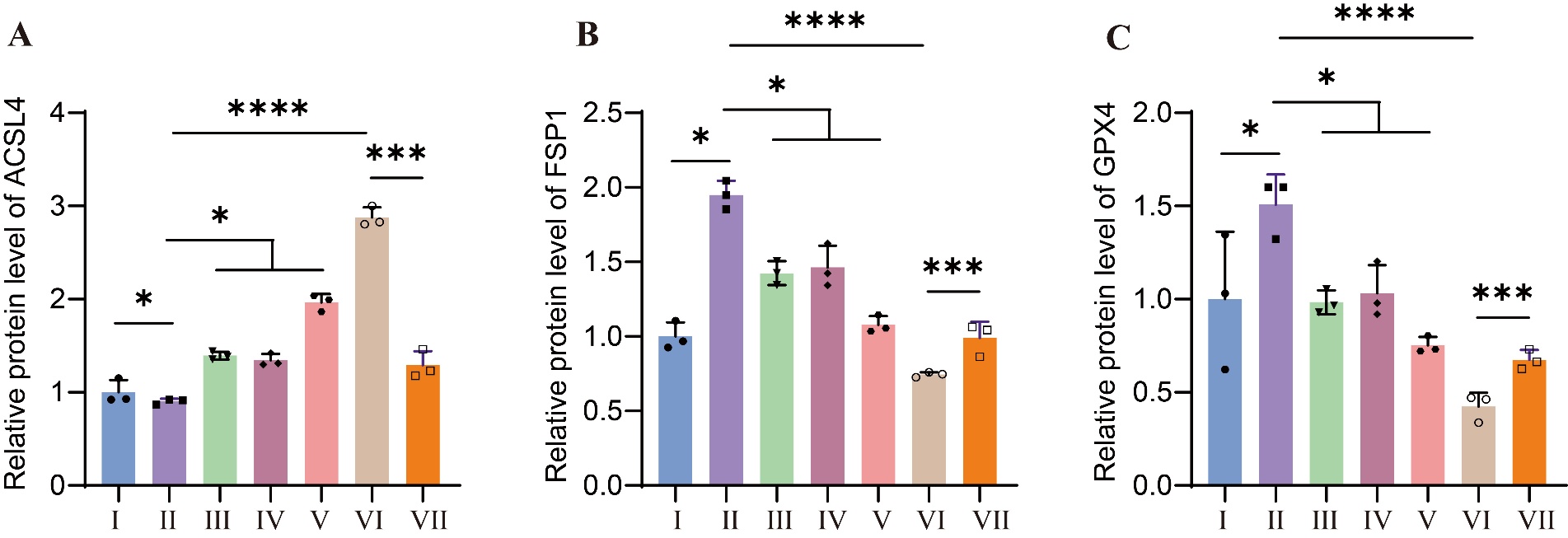


**Figure S11. (A, B, C)** Effects of PBS, *P.a*, *P.a* + Linco, *P.a* + Fe-zyme, *P.a* + Linco + Fe-zyme, *P.a* + Linco@Fe-zyme and *P.a* + Linco@Fe-zyme + Fer-1 on the regulation of ferroptosis in tumor cells, with quantification of ACSL4, FSP1, and GPX4 protein levels from Western blot results. ** P* < 0.05, **** P* < 0.001, ***** P* < 0.0001, ns = no significant difference. Ⅰ: PBS, Ⅱ：*P.a*, Ⅲ: *P.a*+Linco, Ⅳ: *P.a* + Fe-zyme, Ⅴ: *P.a* + Linco + Fe-zyme, Ⅵ: *P.a* + Linco@Fe-zyme, Ⅶ: *P.a* + Linco@Fe-zyme + Fer-1


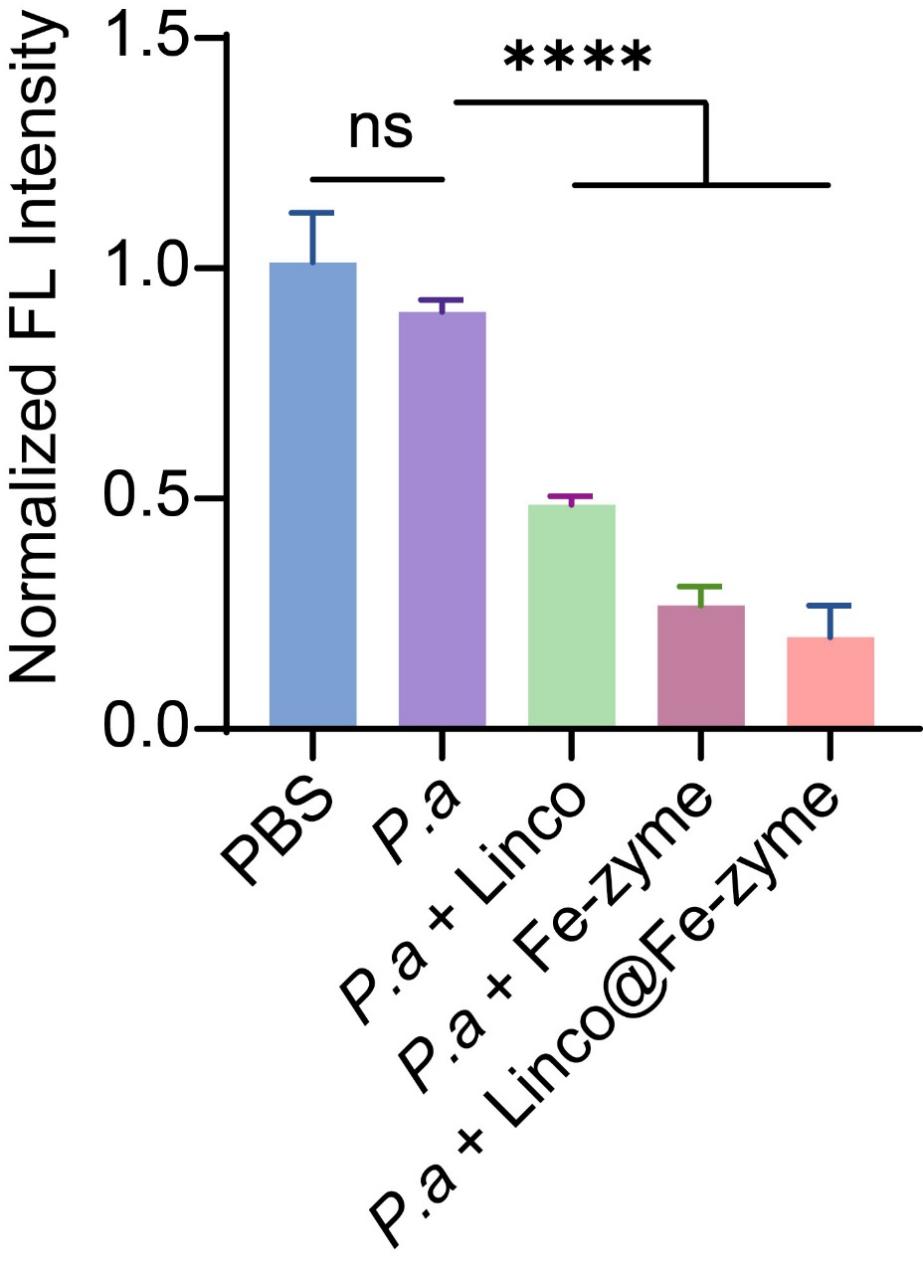


**Figure S12.** Quantitative analysis of HMGB1 fluorescence intensity in LoVo cells. ***** P* < 0.0001, ns = no significant difference.
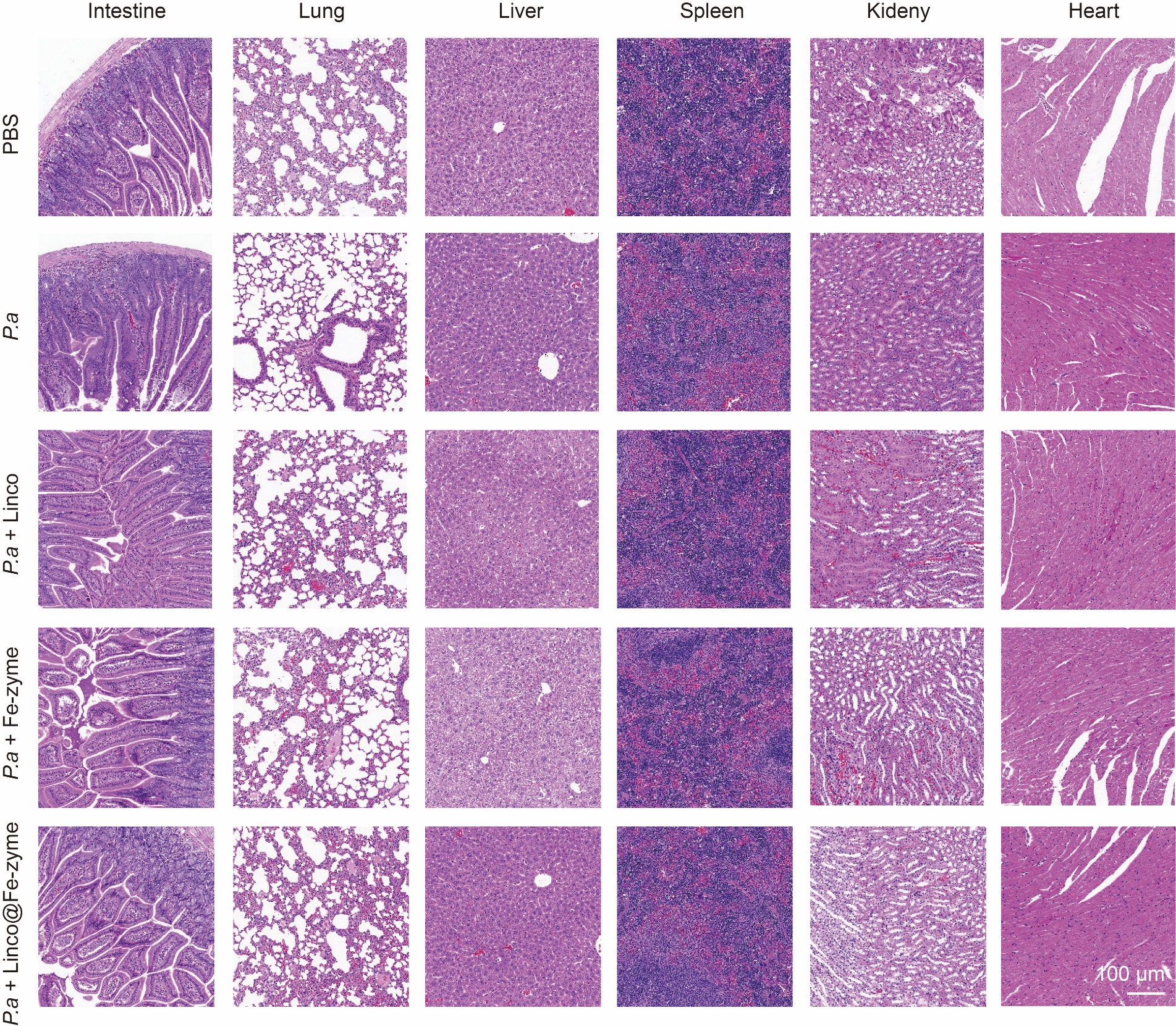


**Figure S13.** H&E staining of intestinal, lung, liver, spleen, kidney, and heart tissues from mice in five treatment groups: PBS, *P.anaerobius*, *P.anaerobius* + Linco, *P.anaerobius* + Fe-zyme, and *P.anaerobius* + Linco@Fe-zyme. The results indicate no significant toxicity among the groups. Scale bar: 50 μm.


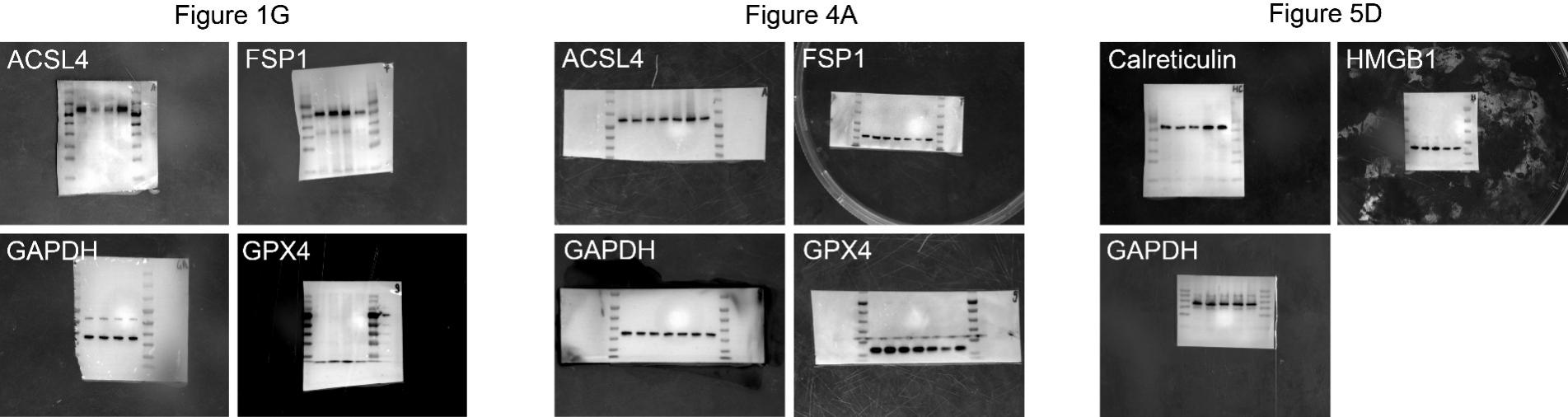


**Figure S14.** The uncropped full-length blots of Figure 1G, 4A, and 5D.
